# Absolute Stereochemistry and Cytotoxic Effects of Vismione E from Marine Sponge-Derived Fungus *Aspergillus* sp. 1901NT-1.2.2

**DOI:** 10.3390/ijms24098150

**Published:** 2023-05-02

**Authors:** Elena V. Girich, Phan Thi Hoai Trinh, Liliana E. Nesterenko, Roman S. Popov, Natalya Yu. Kim, Anton B. Rasin, Ekaterina S. Menchinskaya, Aleksandra S. Kuzmich, Ekaterina A. Chingizova, Artem S. Minin, Ngo Thi Duy Ngoc, Tran Thi Thanh Van, Ekaterina A. Yurchenko, Anton N. Yurchenko, Dmitry V. Berdyshev

**Affiliations:** 1 G.B. Elyakov Pacific Institute of Bioorganic Chemistry, Far Eastern Branch of the Russian Academy of Sciences, Prospect 100-Letiya Vladivostoka, 159, Vladivostok 690022, Russia; 2 Department of Marine Biotechnology, Nhatrang Institute of Technology Research and Application, Vietnam Academy of Science and Technology, Nha Trang 650000, Vietnam; 3 Institute of High Technologies and Advanced Materials, Far Eastern Federal University, 10 Ajax Bay, Russky Island, Vladivostok 690922, Russia; 4 M.N. Mikheev Institute of Metal Physics of the Ural Branch of the Russian Academy of Sciences, S. Kovalevskoi, 18, Ekaterinburg 620108, Russia; 5 Institute of Natural Sciences and Mathematics, The Ural Federal University Named after the First President of Russia B. N. Yeltsin, Lenina Av., 51, Ekaterinburg 620083, Russia

**Keywords:** *Aspergillus*, marine-derived fungus, vismione E, HPLC MS, secondary metabolites, cytotoxicity, MCF-7, proliferation

## Abstract

The metabolic profile of the *Aspergillus* sp. 1901NT-1.2.2 sponge-associated fungal strain was investigated using the HPLC MS technique, and more than 23 peaks in the HPLC MS chromatogram were detected. Only two minor peaks were identified as endocrocin and terpene derivative MS data from the GNPS database. The main compound was isolated and identified as known anthraquinone derivative vismione E. The absolute stereochemistry of vismione E was established for the first time using ECD and quantum chemical methods. Vismione E showed high cytotoxic activity against human breast cancer MCF-7 cells, with an IC_50_ of 9.0 µM, in comparison with low toxicity for normal human breast MCF-10A cells, with an IC_50_ of 65.3 µM. It was found that vismione E inhibits MCF-7 cell proliferation and arrests the cell cycle in the G1 phase. Moreover, the negative influence of vismione E on MCF-7 cell migration was detected. Molecular docking of vismione E suggested the IMPDH2 enzyme as one of the molecular targets for this anthraquinone derivative.

## 1. Introduction

For the last two decades, marine fungi have been promising sources of bioactive compounds [1,2,3]. In particular, marine sponge-derived fungi have excellent potential for the discovery of anticancer agents [4,5].

According to estimates from World Health Organization data, cancer is still one of the leading diseases behind mortality in humans. Worldwide, an estimated 19.3 million new cancer cases and almost 10.0 million cancer deaths occurred in 2020. Furthermore, among women, the most-diagnosed cancer is breast cancer (24.5% of total cases among women), and it is also the biggest cause of mortality among women with cancer, while among men, prostate cancer leads as the most-diagnosed disease (14.1% of total cases among men) [6]. The number of newly diagnosed breast cancer cases is expected to increase by more than 40% by 2040, reaching around 3 million cases annually. In addition, breast cancer mortality is predicted to rise by more than 50%, from 685,000 in 2020 to 1 million in 2040 [7]. Accordingly, new anti-cancer and cancer-preventive drugs will become more and more needed, and marine sponge-derived fungi may be used for this purpose [8].

Previously, during an ongoing research project, a number of marine filamentous fungi were isolated from various sponge samples collected in Nha Trang Bay (Vietnam) [9]. The fungal strain 1901NT-1.2.2 was identified as *Aspergillus* sp., with similarity to *A. europaeus* near 94% and *A. dimorphicus* near 86%. Both these fungi are in the section *Cremei* [10]. The fungi of the *Cremei* section (*Aspergillus brunneouniseriatus*, *A. dimorphicus*, *A. flaschentraegeri*, *A. gorakhpurensis*, *A. itaconicus*, *A. pulvinus*, *A. stromatoides*, *A. wentii*, *Chaetosartorya cremea*, *C. chrysella*, *C. stromatoides,* and others) produce the mycotoxins sterigmatocystin and patulin [11] as well as wentilactones [12], emodin [13], citraconic anhydride, and bianthrones [14], which have anticancer properties [15]. The marine sponge-derived *A. europaeus* was found to be a producer of a number of xanthone-related polyketides [16]. Additionally, various anthraquinone derivatives are one of the most common classes of *Aspergillus* spp. metabolites [17]. Most of them not only have obvious antioxidant properties but also exhibit antitumor effects [18].

The EtOAc extract of the 1901NT-1.2.2 fungal strain culture showed significant cytotoxic activity against human cervical HeLa and breast MCF-7 cancer cells in screening tests, but no information about its cytotoxic secondary metabolites is available.

For this reason, the aims of this work were determination of the metabolite profile of the EtOAc extract of the marine sponge-associated fungal strain *Aspergillus* sp. 1901NT-1.2.2 and isolation of its main individual compounds, as well as studying its cytotoxic activity. Herein, we report on the metabolic profile of *Aspergillus* sp. 1901NT-1.2.2 and the very first isolation from fungi and absolute stereostructure elucidation of known plant anthraquinone vismione E (**1**). Moreover, the cytotoxic activity of vismione E is described in detail for the first time, and its suggested molecular targets are discussed.

## 2. Results

### 2.1. Metabolite Profile of Aspergillus *sp.* 1901NT-1.2.2

**Figure 1:**
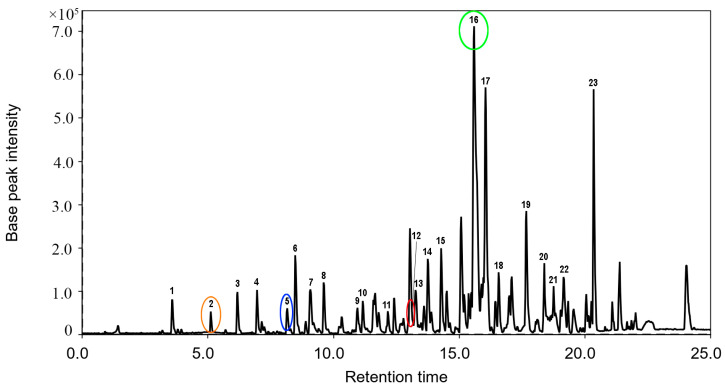
The HPLC MS profile of EtOAc extract of *Aspergillus* sp. 1901NT-1.2.2 fungal strain. Peak #16 corresponds to vismione E (**1**, green circle), peak #2 corresponds to 7-hydroxy-3-(2-hydroxypropyl)-5-methylisochromen-1-one (orange circle), peak #5 corresponds to endocrocin (blue circle), peak #12 corresponds to 11a-hydroxy-4,4,9-trimethyl-9-vinyl-1,2,3,4,9,10,11,11a-octahydrodibenzo[c,e]oxepine-5,7-dione (red circle).

The EtOAc extract of the *Aspergillus* sp. 1901NT-1.2.2 fungal strain was investigated using the HPLC MS technique, and more than 20 peaks were detected (Figure 1).

The peak #2 detected at 5.12 min with *m*/*z* 235.0970 ([M + H]^+^) corresponded to the molecular formula C_13_H_14_O_4_, which was the same as the known fungal isocoumarine polyketide 7-hydroxy-3-(2-hydroxypropyl)-5-methylisochromen-1-one [19]. This was proven through the comparison of experimental MS/MS spectra with the GNPS database (MQScore 0.89). The peak #**5** detected at 8.16 min with *m*/*z* 315.0504 ([M + H]^+^) corresponded to the molecular formula C_16_H_10_O_7_, which was the same as wide-spread anthraquinone derivative endocrocin [20]. This was proven through the comparison of experimental MS/MS spectra (Figure S23) with the GNPS database (MQScore 0.93). The peak #**12** detected at 13.16 min with *m*/*z* 317.1750 ([M + H]^+^) corresponded to the molecular formula C_19_H_24_O_4_. It was identified as 11a-hydroxy-4,4,9-trimethyl-9-vinyl-1,2,3,4,9,10,11,11a-octahydrodibenzo[c,e]oxepine-5,7-dione through the comparison of the experimental MS/MS spectra (Figure S24) with the GNPS database (MQScore 0.83).

For the other 19 peaks, only molecular formulas corresponding to the exact masses were determined (Appendix A, Table A1).

### 2.2. Isolation of Compound ***1***

For the detailed investigation of the chemical composition of the EtOAc extract of the *Aspergillus* sp. 1901NT-1.2.2 fungal strain, it was fractionated using column chromatography, which yielded individual compound **1**.

**Figure 2:**
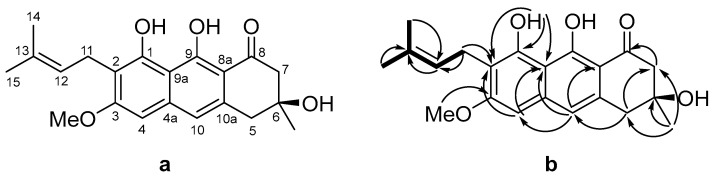
Structure of vismione E (**a**); the key HMBC (arrows) and ^1^H-^1^H COSY (bold lines) correlations (**b**).

The molecular formula of compound **1** (Figure 2a) was determined as C_21_H_24_O_5_ based on the HRESIMS spectrum data containing the [M + Na]^+^ peak at *m*/*z* 379.1515 (Supplementary Materials, Figures S10 and S11), which was confirmed using ^13^C NMR data. The ^1^H and ^13^C NMR spectra contained signals of four methyl groups (including one methoxy group), three methylene groups, three methine groups belonging to sp^2^-carbon atoms, nine quaternary sp^2^-carbon atoms, and one quaternary sp^3^-carbon atom. The analysis of HMBC data, as well as ^1^H-^1^H COSY correlations (Figure 2b, Table 1, Supplementary Materials, Figures S1–S7) allowed us to establish the structure of the prenylated anthraquinone derivative **1** as 3,8,9-trihydroxy-6-methoxy-3-methyl-7-(3 -methylbut-2-en-1-yl)-3,4-dihydroanthracene-1(2H)-one.

The literature data analysis showed that the planar structure of compound **1** was identified as a that of known compound vismione E, which was isolated for the first time from berries of *Psorospermum febrifugum* occurring in Africa and described in the literature only three times as a metabolite of the highest plants [21,22,23,24]. This is the first case of vismione E isolation from a marine microorganism.

The MS data of vismione E (**1**) fully corresponded to those for main peak **#16** in the HPLC MS chromatogram (Figure 1, Table A1). This allows us to consider vismione E as one of the main metabolites of the fungus *Aspergillus* sp. 1901NT-1.2.2.

Analyzing the literature data on vismione E (**1**) showed the absence of any information on either the stereochemistry of this compound or the experimental data used to characterize the stereoconfigurations (CD spectrum, specific optical rotation angle, etc., Supplementary Materials, Figures S8 and S9). In this regard, the problem of determining the configuration of the only chiral center of compound **1** is topical.

**Figure 3:**
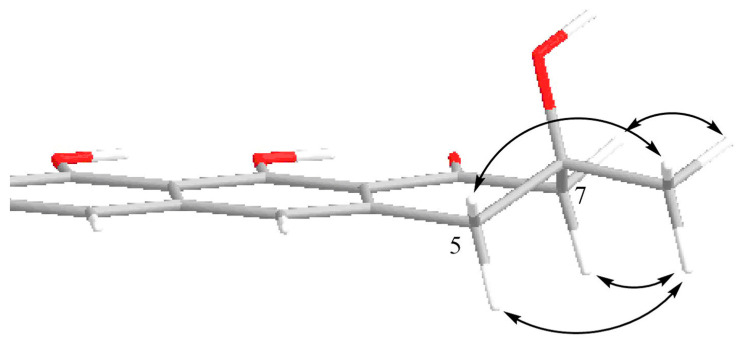
Suggested partial 3D structure of vismione E (**1**) with numbering of key atoms and key ROESY correlations (arrows).

All protons in the aliphatic part of the vismione E molecule were separated, which did not allow us to analyze the vicinal spin–spin interaction constants to establish the stereochemical features of compound **1**. The ROESY correlations between the methyl group at C-6 (δ_H_ 1.44) and all protons of both methylene groups at C-5 (δ_H_ 3.02, 3.06) and C-7 (δ_H_ 2.82, 2.85) indicated the equatorial location of the methyl group. Thus, the relative stereostructure of vismione E (**1**) was assumed, as is shown in Figure 3.

To determine the absolute configuration of compound **1**, we used the ECD spectroscopy method. The description of the used procedure is shown in the Supplementary Materials (Figure S13). The extended quantum-chemical investigation of the UV spectrum and the rotatory powers of different vertical electronic transitions was carried out via the TDDFT approach using different exchange–correlation functionals, implemented in GAUSSIAN 16 software [25]. To overcome the difficulties in the interpretation of experimental ECD spectra in some frequency regions, the evolutions of the ECD spectra along the large amplitude motions (LAM) were investigated (Supplementary Materials, Figures S13–S15).

**Figure 4:**
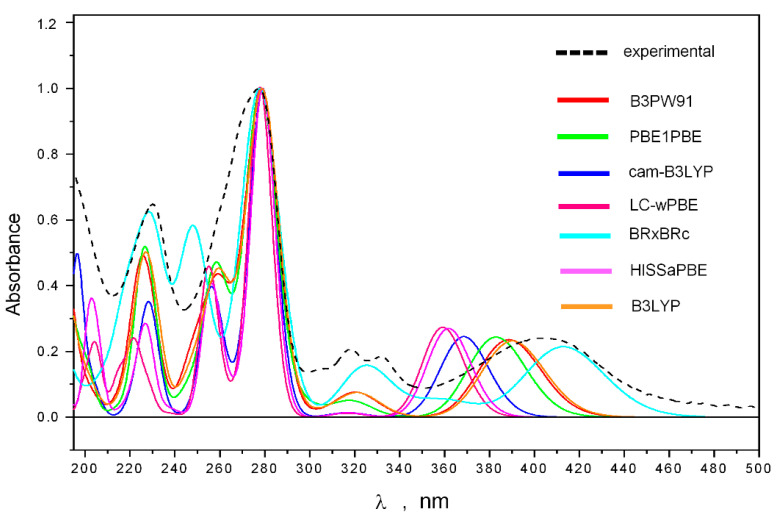
The experimental and theoretical UV spectra of **1**.

The comparison of theoretical (calculated using various density functionals and the 6-311G(d) basis set) and experimental UV spectra is shown in Figure 4. We found that the features of the experimental UV spectrum are well reproduced theoretically in the frequency diapason λ ≤ 340 nm (when using UV shifts, specific for each functional, Supplementary Materials, Table S14).

**Figure 5:**
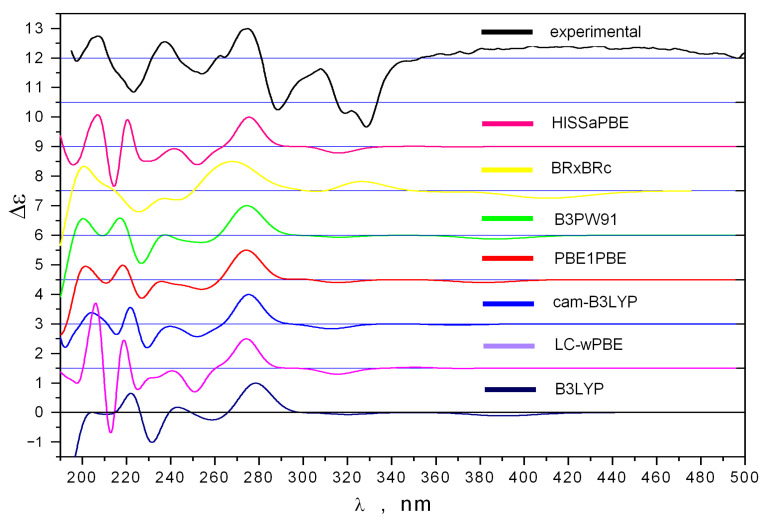
The experimental and theoretical ECD spectra of **1**.

The comparison of experimental and theoretical ECD spectra is shown in Figure 5.

The influence of LAM motions on the shape of the calculated ECD spectra is shown in Supplementary Materials, Figure S16. The main contribution to the total statistically averaged ECD spectrum gives conformations of the “EQ” type—their total amount is nearly twice the number of “AX”-type conformations.

The experimental ECD spectrum of **1** is very complicated. Most bands have sharp shapes. We found that the intensities of negative bands in the 196 ≤ λ ≤ 350 nm region are the strong functions of the LAM1 motion, corresponding to the inversion of ring C. The inversion of this ring may proceed via overcoming the potential energy barriers ΔE^≠^ ≈ 7.4 kcal/mol, associated with two different transition states; each of them has a very nonlinear and even twisted structure (Supplementary Materials, Figure S16). The equilibrium structures, corresponding to minimums on the potential energy surface, have classical envelope shapes. The transfer of **1** from one equilibrium state to another equilibrium state may be described as a movement along the intrinsic reaction coordinate paths (IRC), calculated from TS to minimums on PES. We found that most of these IRC paths for the energy range E ≥ E_min_ + 0.5 kcal/mol are formed with very distorted structures (Supplementary Materials, Figure S17). Hence, the averaged intensities of bands in the 195 ≤ λ ≤ 350 nm region must be, to some extent, other than the intensities obtained for the equilibrium structures.

The comparison of ECD spectra (also for selected individual conformations and for statistically averaged total spectra), calculated using different density functionals, showed that the most variations in the theoretical spectra appear in the λ≤ 240 nm region (Figure 5). This is due, in some ways, to the precision with which different density functionals describe the UV spectrum in this energy diapason. The energy of the first electronic transition is always overestimated by all DFT methods.

The intensity of the band (positive for *S*-**1** and negative for *R*-**1**) at λ ≈ 280 nm is strongly dependent on the LAM2 motion, corresponding to the internal rotation of the OMe group around the C-C bond (Supplementary Materials, Figures S14–S18). However, the position and the sign of this band in the statistically averaged ECD spectrum do not change along the LAM2 motion.

**Figure 6:**
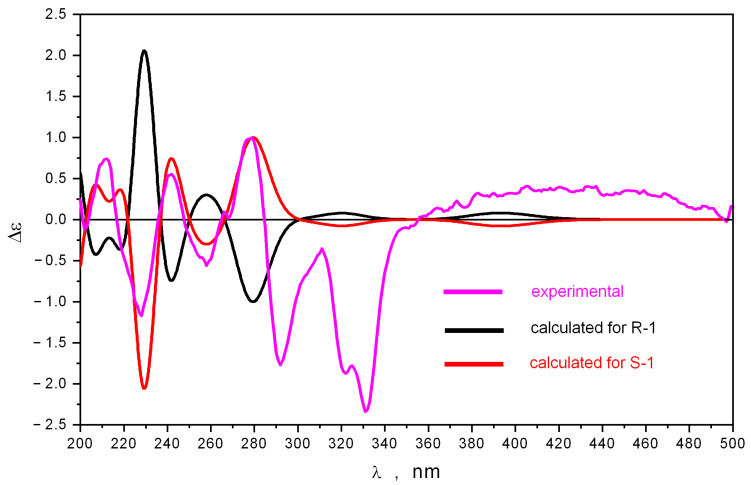
The experimental ECD spectrum of **1** compared to theoretical ECD spectra, calculated using B3PW91/6-311++G(d)_PCM method for stereoisomers 6*S*-1 and 6*R*-1.

These data highlight that the ECD spectrum, averaged over the intramolecular dynamics of **1**, must be different from the ECD spectrum to some extent, calculated as a statistical average of data obtained for stable configurations of **1** only. However, the averaging of ECD spectra over LAM motions is a very complicated task—it requires constructing a correct theoretical (multidimensional) model and strays outside of the purposes of the present work. There is one energy region in which the properties of the ECD spectrum (the signs and positions of bands) do not change under the influence of LAM motions—the 230 ≤ λ ≤ 350 nm region. All DFT methods describe the shape of the ECD spectrum in this region in one and the same manner. Therefore, we selected this region as a reference region in our determination of the stereochemistry of **1** (Figure 6).

The comparison of the ECD spectra calculated for 6*S*-1 and 6*R*-1 demonstrates that a good correspondence to the experimental ECD spectrum occurs for 6*S*-1. The ECD spectrum calculated for 6*R*-1 describes the experimental one very poorly. This allows us to determine the absolute structure of **1** as 6*S*.

### 2.3. Cytotoxic Activity of Vismione E *(****1****)*

#### 2.3.1. Influence on Cell Viability

The cytotoxic activity of vismione E (**1**) was evaluated toward human prostate cancer PC-3 cells and human breast cancer MCF-7 cells, as well as normal human breast epithelial MCF-10A cells and rat cardiomyocytes H9c2. The half-maximal concentrations of vismione E for all used cell lines after 48 h of treatment are presented in Table 2.

**Figure 7:**
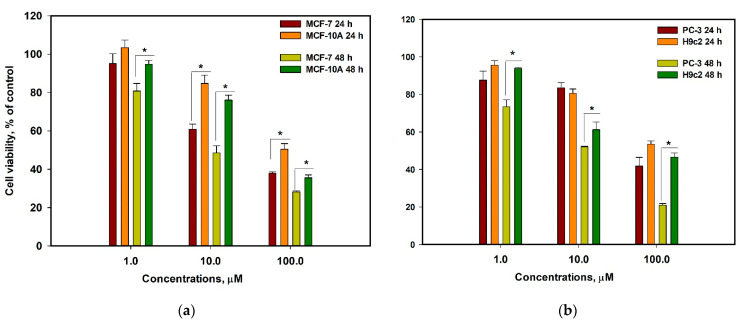
The effect of vismione E (**1**) on cell viability after 24 h and 48 h incubation at different concentrations. (**a**) Effect of vismione E on human breast cancer MCF-7 and normal breast epithelial MCF-10A cells; (**b**) effect of vismione E on human prostate PC-3 and rat normal cardiomyocytes H9c2 cells. The data are presented as mean ± SEM. All experiments were carried out in triplicate. * indicates statistically significant differences between variants (*p* < 0.05).

The effects of vismione E (**1**) on MCF-7 and MCF-10A cell viability as well as PC-3 and H9c2 cell viability after 24 h and 48 h incubation at different concentrations are graphed in Figure 7.

Therefore, vismione E (**1**) showed enhancement of its cytotoxic effect during prolonged administration and more pronounced activity against cancer PC-3 and especially MCF-7 cells than against normal H9c2 and MCF-10A cells.

#### 2.3.2. Influence on MCF-7 Cell Cycle and Proliferation

The influence of vismione E at a concentration of 10 µM on the MCF-7 cell cycle and cell proliferation was investigated after 48 h of treatment.

**Figure 8:**
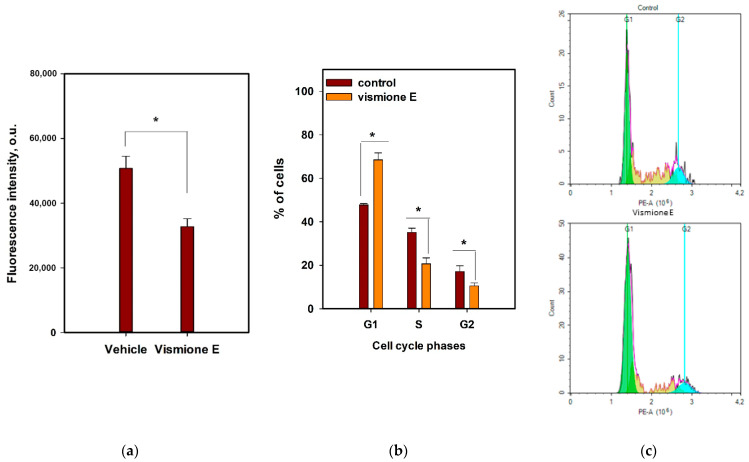
The effect of vismione E (**1**) at 10 µM on (**a**) MCF-7 cell proliferation and (**b**,**c**) MCF-7 cell cycle after 48 h of treatment. The data are presented as mean ± SEM. All experiments were carried out in triplicate. * indicates statistically significant differences between variants (*p* < 0.05).

The influence of vismione E on the MCF-7 cell cycle was investigated using propidium iodide (PI) labeling and detected using the flow cytometry technique (Figure 8b,c). It was found that compound **1** arrested the MCF-7 cell cycle in the G1 phase because the percentage of G1-phase cells in the control was 47.7%, while it was 68.5% in the vismione E-treated cell population. Additionally, the percentage of S- and G2-phase cells was 20.7% and 10.6% in the vismione E-treated cell population, while it was 35.1% and 17.1% in the control, respectively.

The 5-Ethynyl-2′-deoxyuridine (EdU) incorporating assay is based on the property of active proliferating cells including the synthetic thymidine derivative in newly synthesized DNA [26]. The MCF-7 cells after incubation with investigated compound **1** for 48 h were stained with EdU for 2 h, then intracellular EdU was click-reacted with a fluorescent dye and the intensity of fluorescence was measured (Figure 8a). Vismione E decreased the incorporation of EdU into MCF-7 cells by more than 30% in comparison with the non-treated cells.

Moreover, the proliferation of MCF-7 cells was investigated using (5,6)-carboxyfluorescein succinimidyl ester (CFDA SE) staining. The cells were labeled with non-fluorescent CFDA SE dye, which is modified by intracellular esterase enzymes in fluorescent CFDA bonded covalently with amines. The active proliferated cells divide the intracellular reagent between two daughter cells so that the fluorescence intensity in the divided cells decreases compared to the parent or undivided cells [27].

In this work, the distribution of CFDA fluorescence in MCF-7 cells was evaluated using the flow cytometry technique after 48 h of incubation with vismione E in comparison with non-treated cells.

**Figure 9:**
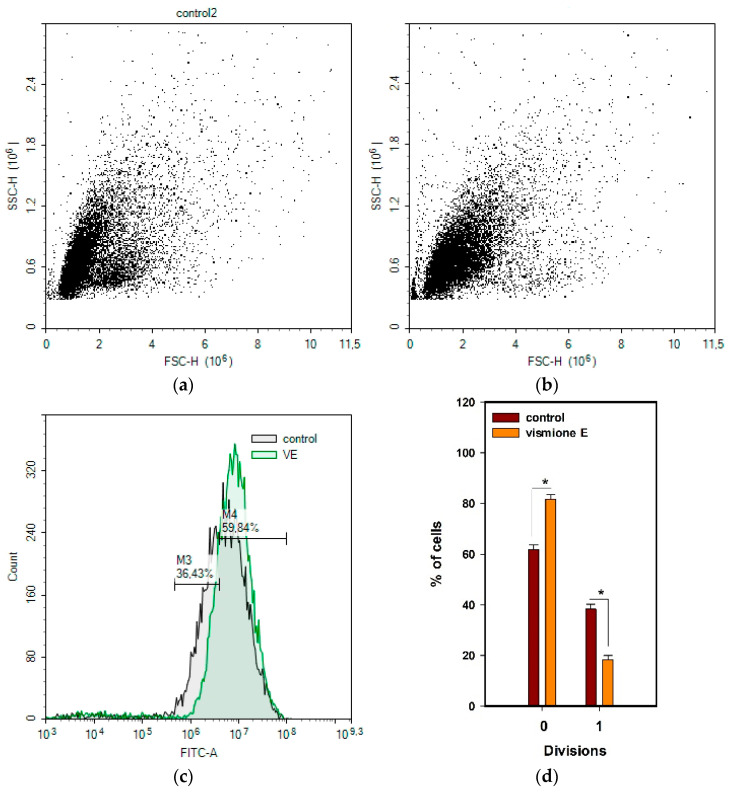
The effect of vismione E (**1**) at 10 µM on MCF-7 cell proliferation. (**a**) Control and (**b**) vismione E-treated MCF-7 cells after 48 h of treatment. (**c**) Merge, (**d**) the percentage of cells in both divisions. The data are presented as mean ± SEM. All experiments were carried out in triplicate. * indicates statistically significant differences between variants (*p* < 0.05).

As a result, the cells were found to undergo two divisions. No division (undivided) showed cells with higher CFDA fluorescence intensity (Figure 9). The cells treated with vismione E showed a significant decrease in the average number of cells in the first division (Figure 9d).

All the obtained data confirmed that vismione E (**1**) suppresses the proliferation of MCF-7 cells and arrests the cell cycle in the G1 phase.

#### 2.3.3. Influence on Cell Migration

**Figure 10:**
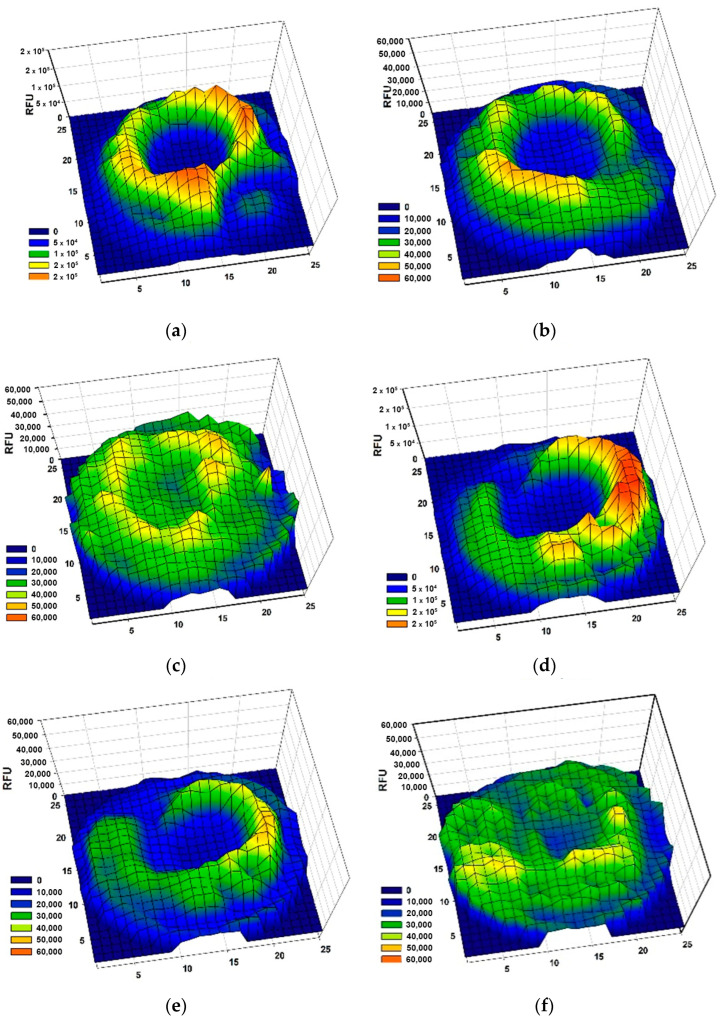
The effect of vismione E (**1**) at 1 µM on MCF-7 cell migration: a fluorescence profile of wells with non-treated cells at (**a**) 0 h, (**b**) 24 h, and (**c**) 96 h after removing the blocker, and the cells treated with **1** at (**d**) 0 h, (**e**) 24 h, and (**f**) 96 h after removing the blocker. The wells were scanned with a matrix with 25 × 25 points. All experiments were carried out in triplicate and the data of one representative well were graphed.

The influence of vismione E at a concentration of 1 µM on MCF-7 cell migration was investigated. Silicon blockers (Figure 15) were inserted into the wells of 24-well plates and the cell suspension was added to each well (Figure S12). After adhesion, the silicon inserts were removed, and MCF-7 cells were stained with CFDA SE for tracking with a plate spectrofluorometer. Then, vismione E at 1 µM or DMSO as a vehicle was added to the wells, and the cells were incubated in the usual conditions. The visualization of MCF-7 migration in the control and vismione E wells by scanning fluorescence intensity on a 25 × 25 points matrix is presented in Figure 10.

**Figure 11:**
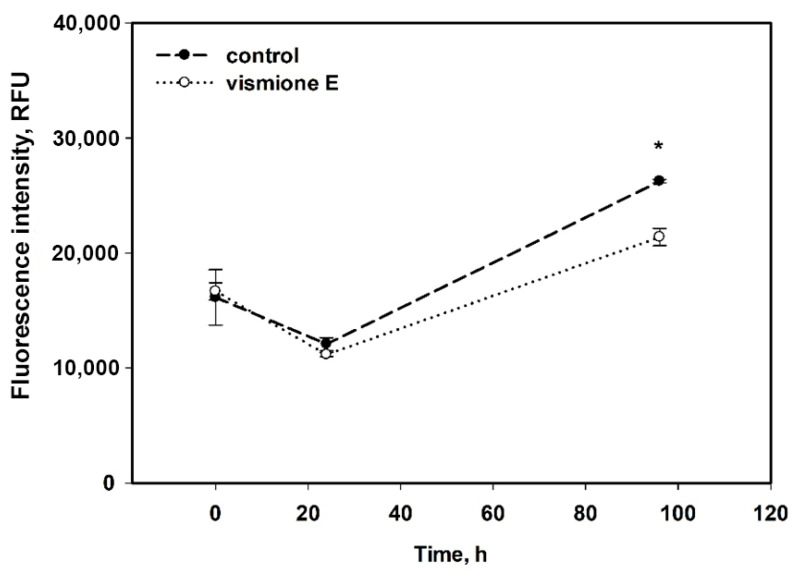
The influence of vismione E at 1 µM on migration of MCF-7 cells for 96 h. All experiments were carried out in triplicate. The data are presented as mean ± SEM. * indicates statistically significant differences between variants (*p* < 0.05).

The average fluorescence intensity in 7 × 7-point central zones is presented in Figure 11. The CFDA fluoresce intensity indicated the number of MCF-7 cells in the central zone of the well for 96 h cultivation, and it was not as intense in the cells treated with vismione E as it was in the control cells.

Therefore, the MCF-7 cells did not migrate quickly, but the control cells filled the well after 96 h of incubation. The cells treated with vismione E remained viable since the fluorescence intensity remained at the control level, but the center of the well remained freer after 96 h of cultivation.

#### 2.3.4. Prediction of Molecular Targets and Molecular Docking

The SwissTargetPrediction web server (http://www.swisstargetprediction.ch/index.php (accessed on 15 February 2023) was used for the prediction of vismione E (**1**) molecular targets [28]. The unique engine behind SwissTargetPrediction calculates the similarity between the user’s query compounds and those compiled in collections of known actives in experimental binding assays.

The list of the top 50 predicted molecular targets is presented in Appendix B (Table A2). The top five of them include inosine-5′-monophosphate dehydrogenase 2 (IMDH2), matrix metalloproteinase 1 (MMP1), ADAM17, complex cyclin C with cell division protein kinase 8 (CCNC/CDK8), and cell division protein kinase 8 (CDK8).

Vismione E was docked with the top five predicted molecular targets to evaluate their binding affinities and active binding residues using the SwissDock docking server (http://www.swissdock.ch (accessed on 15 February 2023)). Docking parameters such as full fitness (FF, kcal/mol), Gibb’s free energy (Δ*G*), and hydrogen bonding (H-bond) were analyzed using UCSF Chimera 1.16 software (Table 3).

All the output clusters of vismione E with the targets were ranked by hydrogen bonding (interactions) and the FF score. A greater negative showed more favorable binding modes with a better fit [29]. Therefore, the docked complex of IMPDH2 with vismione E with the lowest ΔG (−7.478466 kcal/mol) has one H-bond between the Lys229 residue and keto-group at C-8, while another complex with ΔG (−7.4588733 kcal/mol) has two H-bonds between the Lys489 residue and oxygen of the 3-OMe group and the Glu487 residue and the 6-OH group. All complexes have similar lowest FF scores (−5339.8955 kcal/mol and −5338.026 kcal/mol, respectively), but different energies.

The docked complex of vismione E with MMP1 with one H-bond between the Gln974 residue and OH-group at C-6 has the biggest ΔG value (−6.889332 kcal/mol) and a low FF score (−4766.2676 kcal/mol).

The docked complex of vismione E with ADAM17 has the lowest ΔG value (−7.782852 kcal/mol), but the highest FF score (−3054.5269 kcal/mol) despite the four H-bonds calculated between the Ala270 and Lys 273 residues and the 6-OH group, Ala266 residue and keto-group at C-8, and Lys455 residue and OH-group at C-9.

The docked complex of vismione E with CDK8CCNC has a good ΔG value (−7.634472 kcal/mol), but only one H-bond between the Lys18 residue and OH-group at C-9, and a high FF score (−3557.7717 kcal/mol).

**Figure 12:**
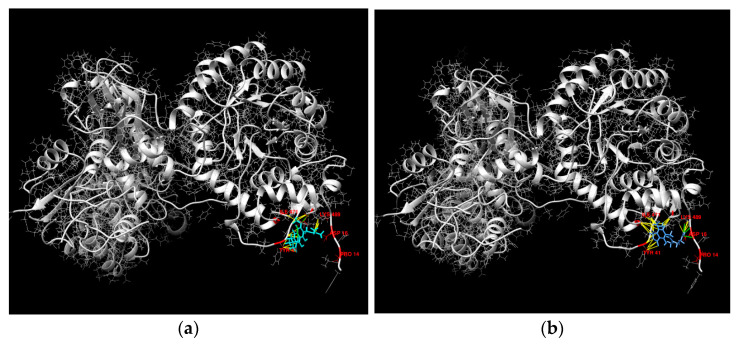

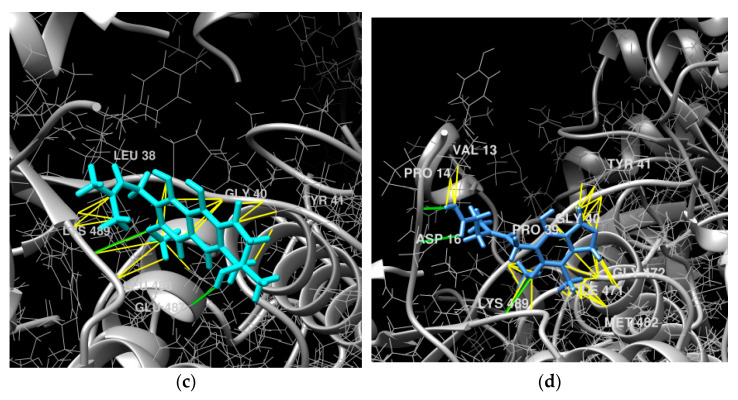
The molecular docking of vismione E (**a**,**c**) and MPA (**b**,**d**) complex with IMPDH2. The calculated hydrogen bonds are green and hydrophobic interactions are yellow.

Therefore, the interaction of vismione E with IMPDH2 may be more energy-efficient. To compare our data on the interaction of vismione E with this molecular target with a known inhibitor of IMPDH2 activity, its complex with mycophenolic acid (MPA) was docked (Figure 12). The IMPDH2 complex with MPA has a ΔG value of −7.7401905 kcal/mol and three H-bonds between the Lys489 residue and OMe-group of MPA, Pro14 residue and carboxy OH-group, and Asp16 residue and carboxy carbonyl group of the MPA structure. The FF score was calculated as −5380.3955 kcal/mol. Thus, vismione E may interact with the Lys489 residue of IMPDH2 like MPA, and the complex IMPDH2/vismione E has similar FF scores.

To analyze the influence of the prenyl group on vismione E’s possible interaction with IMPDH2, the complex of a theoretical de-prenylated derivative of vismione E with IMPDH2 was docked. One complex with a H-bond between the Lys489 residue and oxygen of the 3-OMe group (similar to vismione E) was calculated, but its ΔG value (−6.7235613 kcal/mol) was higher than that of the IMPDH2/vismione E complex. The complex with the H-bond between the Asp15 residue and OH-group at C-6 (near the MPA site of interaction) also has a high ΔG value (−6.6762652 kcal/mol). Therefore, the prenyl chain of vismione E has a significant influence on its interaction with the estimated molecular target.

## 3. Discussion

Previously, the EtOAc extract of *Aspergillus* sp. 1901NT-1.2.2 at a concentration of 10 µg/mL significantly decreased the viability of human breast cancer MCF-7 cells by 83.7% [9], and now, the individual compound vismione E with high cytotoxic activity against MCF-7 and PC-3 cancer cells was isolated from this fungal extract.

Therefore, this is the first report about the cytotoxic activity of vismione E, which was found as a main low-weight secondary metabolite of *Aspergillus* sp. 1901NT-1.2.2 strain. Along with this one, 7-hydroxy-3-(2-hydroxypropyl)-5-methylisochromen-1-one, endocrocin, and 11a-hydroxy-4,4,9-trimethyl-9-vinyl-1,2,3,4,9,10,11,11a-octahydrodibenzo[c,e]oxepine-5,7-dione were identified in the EtOAc extract of the *Aspergillus* sp. 1901NT-1.2.2 fungus using the HPLC MS technique. Endocrocin is the well-known polyketide metabolite of fungi, and was reported to be a cyclooxygenase inhibitor [30], glutamate dehydrogenase inhibitor, DPPH scavenger [31], and suppressor of neutrophil migration [32]. The polyketide derivative 7-hydroxy-3-(2-hydroxypropyl)-5-methylisochromen-1-one was previously isolated from the endolichenic fungus *Ulocladium* sp. [19] and endophytic fungus *Alternaria alternata* [33]. Thus, this is the first report about its detection in a marine-derived fungus.

**Figure 13:**
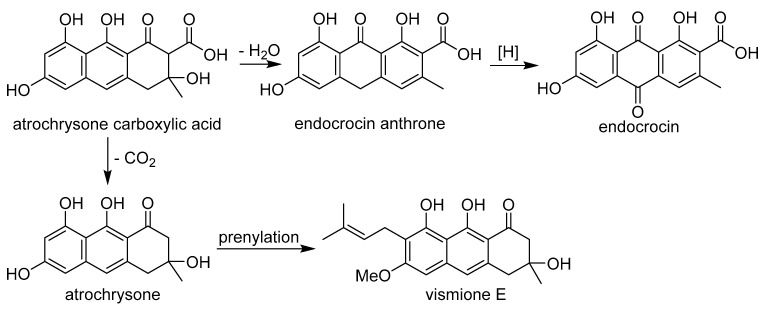
Probable biogenetic relationship between vismione E (**1**) and endocrocin.

The anthraquinone derivatives endocrocin and vismione E found in the fungal extract are clearly formed from the common precursor atrochrysone carboxylic acid (Figure 13), as was previously shown for endocrocin [34]. Unfortunately, we were unable to detect this precursor in the extract. It was previously indicated that it is contained in endocrocin-producing fungi only in trace amounts [35].

The significant effect of vismione E on human breast cancer MCF-7 cell viability was detected using an MTT assay, which is widely used but had some limitations. The decrease in optical density may be caused by the decrease in the number of cells as a result of the inhibition of the proliferation or death of cells in various ways [36]. Therefore, the data on the influence of vismione E on MCF-7 cell viability obtained through the MTT assay should be verified. In our experiments, the influence of vismione E on MCF-7 cell proliferation and arrest of the cell cycle in the G1/S phase was confirmed. The inhibition of proliferation caused a greater cytotoxic effect of vismione E after 48 h of cell treatment in comparison with treatment for 24 h.

Previously, a high cytotoxic effect against MCF-7 cells was found for the other vismione-related compounds vismione B and deacetylvismiones A and H, with a GI_50_ of 4.5 μM, 5.1 μM, and 1.2 μM, respectively, but these data cannot be fully compared with our results because the experimental conditions were not described in detail [22].

The literature databases mention the possibility of vismione E inhibiting GLI1 transcriptional activity, and the authors of this article used vismione E’s chemical structure for pharmacophore modeling, but the initial reference does not have any data about this activity of vismione E [37,38].

The prediction of molecular targets using the SwissTargetPrediction web server is based on the quantification of similarity, which consists of computing a pair-wise comparison of 1D vectors describing molecular structures. The 2D measure uses the Tanimoto index between path-based binary fingerprints (FP2) and the 3D measure is based on a Manhattan distance similarity quantity between Electroshape 5D (ES5D) float vectors. The latter mines five descriptors for each atom of 20 previously generated conformations (Cartesian coordinates, partial charge, and lipophilic contribution). For both the 2D and 3D similarity measures, the principle is that two similar molecules are represented by analogous vectors, which exhibit a quantified similarity close to **1** [39].

**Figure 14:**
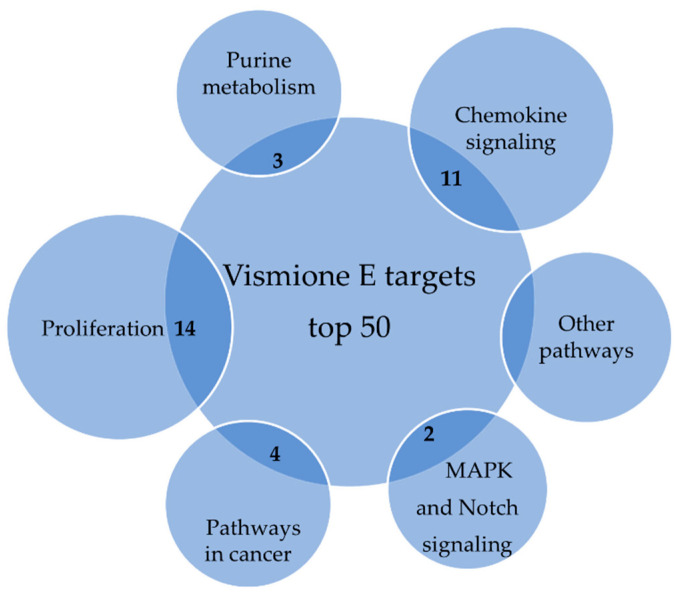
The probable macromolecular targets of vismione E according to SwissTargetPrediction database.

The analysis of possible targets with the KEGG database shows that they are involved in DNA synthesis, proliferation, and chemokine signaling, and several targets each belong to the purine pathway, MAPK and Notch signaling, and specific metabolic pathways of tumor cells (Figure 14).

The top five predicted targets for vismione E include cell proliferation and migration processes as well as specific cancer pathways. CDK8 in complex with cyclin C is a transcriptional regulator that mediates several carcinogenic pathways in breast cancer [40]. The CDK8 submodule can interact directly with transcription factors independently of the mediator complex to regulate signaling pathways including Notch-dependent signaling, transforming growth factor-β (TGF-β) and bone morphogenetic protein (BMP) receptor signaling, and signal transducer and activator of transcription (STAT) signaling, so it is a potential drug target for breast and prostate cancers [41].

MMPs including MMP1 are involved in many biological processes, such as tissue repair and remodulation, cellular differentiation, embryogenesis, morphogenesis, cell mobility, angiogenesis, cell proliferation, and migration [42]. MMPs are used by cancer cells to hydrolyze the structural proteins that comprise the host extracellular matrix for fast invasion and migration. It was shown that MMP-1 cleaves the PAR1 receptor for activation and generates PAR1-dependent Ca^2+^ signals in MCF-7 breast cancer cells and promotes tumorigenesis in a xenograft in vivo model [43]. Another metalloprotease, A disintegrin and metalloprotease 17 (ADAM17), is involved in the processing of the interleukin-6 receptor (IL-6R), the pro-inflammatory cytokine tumor necrosis factor α (TNFα), and most ligands of the epidermal growth factor receptor (EGFR). ADAM17 plays also an important role in breast cancer, where it has been shown to influence cell invasion and proliferation, but also angiogenesis and cancer cell apoptosis [44].

The IMPDH enzyme present in IMPDH1 and IMPDH2 isoforms catalases the conversion of inosine 5′-phosphate (IMP) to xanthosine 5′-phosphate (XMP), the first committed and rate-limiting step in the de novo synthesis of guanine nucleotides, and, therefore, plays an important role in the regulation of cell growth [45].

One of the well-known IMDH2 inhibitors is mycophenolic acid, which was isolated from *Penicillium brevicompactum* fungus many years ago, and its effects on cancer cells include growth inhibition, cell cycle arrest, inhibition of migration, and others [46,47]. After unclear results of phase I clinical trials of MPA against relapsed/refractory multiple myeloma, the investigation of IMDH2 inhibitors as anticancer drug candidates was stopped, and research refocused on targeting the study of immunosuppressive properties. Nonetheless, the study of new IMDH2 inhibitors has resulted in the rediscovery of their anticancer potential [48].

It was found that one of the isoforms of IMPDH, IMPDH1, may be expressed in both normal and cancer cells, but IMPDH2 is expressed preferably in cancer-transformed cells [49,50]. Therefore, the more specific influence of vismione E on cancer cells in comparison to normal cells may be consistent with this fact.

Thus, IMDH2 can be considered as one of the molecular targets for vismione E. However, the successful molecular docking of vismione E with other proteins (ΔG < 0 kcal/mol) shows that this low-molecular weight secondary metabolite may be a multitarget ligand.

## 4. Materials and Methods

### 4.1. General

Optical rotations were measured on a Perkin-Elmer 343 polarimeter (Perkin Elmer, Waltham, MA, USA). UV spectra were recorded on a Shimadzu UV-1601PC spectrometer (Shimadzu Corporation, Kyoto, Japan) in methanol. CD spectra were measured with a Chirascan-Plus CD spectrometer (Leatherhead, UK) in methanol. NMR spectra were recorded on a Bruker DRX-700 spectrometer (Bruker BioSpin GmbH, Rheinstetten, Germany), using TMS as an internal standard. HRESIMS spectra were measured on a Maxis Impact mass spectrometer (Bruker Daltonics GmbH, Rheinstetten, Germany).

Low-pressure liquid column chromatography was performed using silica gel (50/100 μm, Imid Ltd., Krasnodar, Russia). Plates (5 × 10.0 cm) precoated with silica gel (5–17 μm, Imid Ltd., Krasnodar, Russia) were used for thin-layer chromatography. Preparative HPLC was carried out on a Shimadzu LC-20 chromatograph (Shimadzu USA Manufacturing, Canby, OR, USA) using a YMC ODS-AM (YMC Co., Ishikawa, Japan) (5 µm, 10 mm × 250 mm) column with a Shimadzu RID-20A refractometer (Shimadzu Corporation, Kyoto, Japan).

### 4.2. Fungal Material and Fermentation

The isolation and identification of the fungus *Aspergillus* sp. 1901NT-1.2.2 strain were reported previously [9]. The strain is stored in the collection of marine microorganisms of the Nha Trang Institute of Technology Research and Application, VAST (Nha Trang, Vietnam), under code 1901NT-1.2.2 (GenBank accession number MN577307).

The fungus was cultured in 100 × 500 mL Erlenmeyer flasks, each containing rice (20.0 g), yeast extract (20.0 mg), KH_2_PO_4_ (10 mg), and natural seawater from Nha Trang Bay (40 mL) at 28 °C for three weeks.

### 4.3. Extraction and HPLC MS Analysis

The fungal mycelia and medium were extracted with EtOAc (24.0 L) and then evaporated in vacuo to yield a crude extract (20.0 g), to which 250 mL of H_2_O–EtOH (4:1) was added, and the combination was thoroughly mixed to yield a suspension.

HPLC MS analysis was performed using a Bruker Elute UHPLC chromatograph (Bruker Daltonics, Bremen, Germany) connected to a Bruker Impact II Q-TOF mass spectrometer (Bruker Daltonics, Bremen, Germany). An InfinityLab Poroshell 120 SB-C18 column (2.1 × 150 mm, 2.7 μm, Agilent Technologies, Santa Clara, CA, USA) was used for chromatographic separation. The mobile phases were 0.1% formic acid in H_2_O (eluent A) and 0.1% formic acid in MeCN (eluent B). The gradient program was as follows: from 10% to 45% eluent B from 0 to 10 min, from 45% to 100% eluent B from 10 to 20 min, isocratic at 100% of eluent B to 25 min, from 100% to 10% eluent B from 25 to 25.2 min. After returning to the initial conditions, equilibration was achieved after 5 min. Chromatographic separation was performed at a 0.4 mL/min flow rate at 40 °C. The injection volume was 2 μL.

The mass spectrometry detection was performed using an ESI ionization source in positive ion mode. The optimized ionization parameters for ESI were as follows: capillary voltage of 4.5 kV, nebulization with nitrogen at 2.5 bar, dry gas flow of 6 L/min at a temperature of 200 °C. The mass spectra were recorded within the *m*/*z* mass range of 50–2000 (scan time 1 s). Collision-induced dissociation (CID) product ion mass spectra were recorded in auto-MS/MS mode with a collision energy ranging from 15 eV at 100 *m*/*z* to 120 eV at 1500 *m*/*z* (the exact collision energy setting depended on the molecular masses of precursor ions). The precursor ions were isolated with an isolation width of 4 Th.

The mass spectrometer was calibrated using the ESI-L Low Concentration Tuning Mix (Agilent Technologies, Santa Clara, CA, USA). The instrument was operated using the otofControl (ver. 4.1, Bruker Daltonics, Bremen, Germany), and data were analyzed using DataAnalysis Software (ver. 4.4, Bruker Daltonics, Bremen, Germany). 

#### Data Analysis

UHPLC-Q-TOF data were converted from Bruker “.d” formatting to “.mzXML” using MSConvert 3.0 (part of ProteoWizard 3.0 package, Palo Alto, California, USA) [51], and further processing was performed with MZMine (version 2.53) [52]. The MZMine processing settings were as follows: mass detection was carried out at the MS1 level and MS2 level with noise level thresholds of 60 and 40, respectively. Chromatograms were made with the ADAP Chromatogram Builder Module [53] with the following parameters: min group size in # of scans was set to 6, group intensity threshold and min highest intensity were set to 130 and 300, respectively, *m*/*z* tolerance was set to 0.05 *m*/*z*. The chromatogram deconvolution module was used with the ADAP algorithm with a signal/noise threshold of 8, min feature height of 300, and coefficient/area threshold of 40, while peak duration range was set from 0 to 2.0 and RT wavelet range was set from 0 to 0.1. The *m*/*z* center calculation was set to MEDIAN. The Isotopics peaks grouper module was used with an *m*/*z* tolerance of 5 ppm, retention time tolerance of 0.1 min, the monotonic shape function set to true, a maximum charge of 2, and the representative isotope set to the most intense. Alignment was achieved with the Join aligner function with an *m*/*z* tolerance of 5 ppm, a weight for *m*/*z* at 50, a retention time tolerance of 0.1 min, and a weight for RT at 50. The Require same charge state, Require same ID, and the Compare spectra similarity functions were set to false. The aligned feature list was exported using the Export/Submit to “GNPS-FBMN” module with the Merge MS/MS (experimental) function with the following parameters: select spectra to merge was set to across samples, the *m*/*z* merge mode was set to weighted average (remove outliers), the intensity merge mode was set to sum intensities, the expected mass deviation was set to 5 ppm, the cosine threshold was set to 70%, the peak count threshold was set to 20%, the isolation window offset (*m*/*z*) was set to 0, and the isolation window width (*m*/*z*) was set to 3.

Dereplication of the MS/MS spectra was carried out using the GNPS module Library Search. All parameters were maintained as default.

### 4.4. Isolation of Vismione E

The fungal mycelia and medium were extracted with EtOAc (24.0 L) and then evaporated in vacuo to yield a crude extract (20.0 g), to which 250 mL of H_2_O–EtOH (4:1) was added, and the combination was thoroughly mixed to yield a suspension. It was extracted successively with hexane (150 mL × 2), EtOAc (150 mL × 2), and n-BuOH (150 mL × 2). The EtOAc fraction was concentrated in vacuo to give a residue (6.0 g), which was separated on a silica gel column (25 × 3 cm) eluted with a hexane-EtOAc system with 5-% gradient (1:0–0:1). The hexane-EtOAc fraction AFl-1-52 (85:15, 5.1 g) was separated using a sephadex LH-20 column (CHCl_3_–EtOH, 1:1) to yield the fraction AFl-4-35 (166.6 mg), which was purified using an ODS-microcolumn (6 × 1 cm) followed by purification through RP HPLC on a YMC ODS-AM column, eluting with MeOH–H_2_O (65:35) to yield **1** (4.4 mg). The hexane-EtOAc fraction AFl-1-64 (75:25, 2.5 g) was separated using a sephadex LH-20 column eluted with EtOH-CHCl_3_ (1:4) to yield the fraction AFl-23-5 (253.5 mg). Fraction AFl-23-5 was separated through column chromatography on SiO_2_ (12 × 2 cm) eluted with the hexane-EtOAc system (1:0–0:1) to yield the fraction AFl-35-35 (49.1 mg). Fraction AFl-35-35 was purified using an ODS-microcolumn (6 × 1 cm) followed by RP HPLC (grad. from 10% MeCN in water to 100% over *40* min) and further isocratic RP HPLC on a YMC ODS-AM column, eluting with MeCN–H_2_O (50:50) to yield compound **1** (1.5 mg).

#### Vismione E (**1**)

Yellow oil; [α]^20^_D_ +35.3° (c 0.05, MeOH); UV (MeOH) λ_max_ (logε) 404 (3.96), 331 (3.80), 317 (3.87), 277 (4.62), 231 (4.41), 196 (4.41) (Figure S8); CD (0.28 mM, MeOH) λ_max_ (Δε) 212 (+0.43), 228 (–0.68), 242 (+0.32), 258 (−0.33), 267 (+0.04), 279 (+0.58), 292 (−1.03), 311 (−0.20), 322 (−1.09), 331 (−1.36) (Figure S9); ^1^H and ^13^C NMR data, see Supplementary Materials (Figures S1–S7); HRESIMS [M + Na]^+^ 379.1515 (calcd for C_21_H_24_NaO_5_, 379.1516) (Figure S11).

### 4.5. Bioassays

#### 4.5.1. Cell Culture

The human prostate cancer PC-3 and breast cancer MCF-7 cells were purchased from ATCC (Manassas, VA, USA). The rat cardiomyocytes H9c2 cells were kindly provided by Prof. Dr. Gunhild von Amsberg from the Martini-Klinik Prostate Cancer Center, University Hospital Hamburg-Eppendorf, Hamburg, Germany. The normal breast epithelial MCF-10A cells were kindly provided by the Institute of Gene Biology RAS, Moscow, Russia. All cell lines were cultured in DMEM with 10% of fetal bovine serum and 1% of penicillin/streptomycin (BioloT, St. Petersburg, Russia). The PC-3 and MCF-7 cells were seeded at concentrations of 5 × 10^3^ cell/well, and H9c2 and MCF-10A cells were seeded at a concentration of 3×10^3^ cell/well. The experiments were started after 24 h.

#### 4.5.2. Cell Viability Assay

The cells were treated with compounds at concentrations up to 100 µM for 24 h or 48 h, and the viability of cells was measured using an MTT assay.

The in vitro cytotoxicity of individual substances was evaluated using the MTT (3-(4,5-dimethylthiazol-2-yl)-2,5-diphenyltetrazolium bromide) assay, which was performed according to the manufacturer’s instructions (Sigma-Aldrich, St.-Louis, MO, USA). The results are presented as a percentage of control data, and the concentration at 50% cell viability inhibition (IC_50_) was calculated.

#### 4.5.3. EdU Incorporation Assay

The MCF-7 cells were seeded in a 96-well plate and treated with the compound at a concentration of 10 µM for 48 h. After 46 h of incubation, the dH_2_O solution of 5-ethynyl-2′-deoxyuridine (EdU) (Lumiprobe, Moscow, Russia) at a concentration of 10 µM was added to each well for 2 h, and then they were stained in accordance with the instructions of the manufacturer [54]. The cell monolayer was rinsed with phosphate-buffered saline (PBS) three times and permeabilized with 0.2% Triton X-100 (Helicon, Moscow, Russia) in PBS for 1 h at room temperature. Then, the cells were stained through a click reaction with ascorbic acid at 10 mM (Lumiprobe, Moscow, Russia), Cu(II)-TBTA complex at 2 mM (Lumiprobe, Moscow, Russia), and sulfo-Cyanine5 Azide at 8 µM (Lumiprobe, Moscow, Russia) in 100 mM Tris buffer pH 7.4 for 30 min at room temperature in the dark. After that, the cells were washed with PBS twice.

The fluorescence intensity was measured at λ_ex_ = 485 and λ_em_ = 520 nm using the plate reader PHERAstar FS (BMG Labtech, Offenburg, Germany). The data were processed using MARS Data Analysis v. 3.01R2 (BMG Labtech, Offenburg, Germany). The data are presented in relative fluorescent units.

#### 4.5.4. Cell Cycle Investigation

The MCF-7 cells were seeded in a 12-well plate for 24 h and then treated with the compound at a concentration of 10 µM for 48 h.

After incubation, cells were scrabbed, harvested, washed with PBS, and fixed with ice-cold 70% ethanol in a dropwise manner prior to storage at −20 °C overnight. The cells were then washed with PBS and incubated with 200 μg/mL RNAse (PanReac, AppliChem, Germany) and 20 μg/mL of propidium iodide (Sigma-Aldrich, St. Louis, MO, USA) for 30 min at 37 °C, and the DNA content was analyzed with a NovoCyte flow cytometer, (Agilent, Austin, TX, USA). The proportion of cells in each phase of the cell cycle is expressed as a percentage.

#### 4.5.5. CFDA SE Proliferation Assay

The MCF-7 cells were seeded in a 12-well plate for 24 h. After adhesion, the cells were stained with (5,6)-carboxyfluorescein succinimidyl ester (CFDA SE) dye (LumiTrace CFDA SE kit, Lumiprobe, Moscow, Russia). CFDA SE stock solution at 5 mM in DMSO was dissolved in PBS to prepare a 10 µM solution. The cell culture medium was replaced with this CFDA SE solution for 5 min at 37 °C. Then, the cell layer was washed with PBS twice, the cell culture medium was added to each well, and the compound at a concentration of 10 µM was added to each well for 48 h. DMSO at the same concentration was used as a control.

After incubation, the cells were washed with PBS twice, scrabbed, and collected in 1.5 mL tubes. The intensity of CFDA fluorescence was analyzed with a NovoCyte flow cytometer (Agilent, Austin, TX, USA).

#### 4.5.6. Cell Migration Assay

The self-made silicon inserts were placed in the center of the wells in a 24-well plate, and MCF-7 cell suspension was added to each well for 24 h. After adhesion, the inserts were removed and the cells were labeled with (5,6)-carboxyfluorescein succinimidyl ester (CFDA SE) dye (LumiTrace CFDA SE kit, Lumiprobe, Moscow, Russia). CFDA SE stock solution at 5 mM in DMSO was dissolved in PBS to prepare a 10 µM solution. The cell culture medium was replaced with this CFDA SE solution for 5 min at 37 °C. Then, the cells were washed twice with PBS and the fluorescent profile of each well with a 25×25 points matrix was scanned at λ_ex_ = 485 and λ_em_ = 520 nm using the plate reader PHERAstar FS (BMG Labtech, Offenburg, Germany). The data were processed using MARS Data Analysis v. 3.01R2 (BMG Labtech, Offenburg, Germany).

The investigated compound at a concentration of 1 µM was added to each well (DMSO was used as a control), and the cells were incubated at 37 °C. The scanning of the fluorescent profile of the wells was carried out after 24 h and 96 h of incubation. The data were obtained as a relative fluorescent units and visualized in a 3D graph using SigmaPlot software.

#### 4.5.7. Preparation of Suction Cup Inserts for Cell Migration Assay

**Figure 15:**
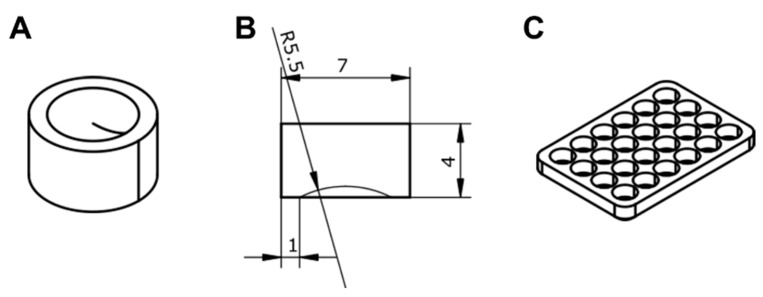
(**A**) Bottom side of the insert, (**B**) key dimensions of the insert (mm), (**C**) sketch of the mold.

To study cell migration, custom silicone inserts were placed in a 24-well plate (Figure 15 and Figure S12). To do this, molds were designed and printed on an MSLA 3D printer, into which two-component silicone was added.

The design of inserts and corresponding molds was carried out in the open-source software freeCAD version 20.1 (www.freecadweb.org (accessed on 10 September 2022)). The molds were designed for 24 inserts to make a full set of plate inserts. A schematic view of a single insert and a mold is shown in the figure. The slicing of the .stl file was carried out in Chitubox 1.9.4 software (CBD Technology LTD, Beijing, China). Printing was carried out using an Anycubic Photon Mono 3D printer (Anycubic. Beijing, China) and Anycubic Basic Translucent Green resin (Anycubic, China). After printing, the molds were washed in dirty isopropyl alcohol, after which they were washed in pure alcohol using an Anycubic Wash & Cure 2.0 (Anycubic, China), then dried with compressed air. Curing was also carried out with the Wash & Cure station for 15 min.

Two-component tin-based cast silicone Elastoform-T (IP Voinova, Russia) was poured into the finished molds. Any other low viscosity two-component silicone would work as well, but only with a tin-based catalyzer. Platinum-catalyzed silicones do not polymerize in the presence of an MSLA resin. After the silicone was poured into the molds, they were evacuated to remove air bubbles. The silicone completely polymerized within 24 h, after which the inserts were carefully removed from the molds with tweezers. Model files, as well as more detailed instructions for making molds and inserts, are available at https://github.com/arteys/WellSucker (accessed on 10 September 2022).

The inserts were sterilized by dipping in 70% ethanol for a few minutes, then dried and dipped in phosphate buffer to completely remove any residual ethanol. After that, the inserts were placed in the middle of the wells of the 24-well plate with tweezers and lightly pressed down so that the insert stuck to the bottom of the plate.

### 4.6. Search of Proposed Molecular Targets of Vismione E

The SwissTargetPrediction database (http://www.swisstargetprediction.ch (accessed on 15 February 2023)) was used to identify the potential targets with the screening criteria of a probability of 0.1. The Kyoto Encyclopedia of Genes and Genomes (KEGG) database (http://www.genome.ad.jp/kegg/ (accessed on 16 February 2023) was used to detect pathways for potential molecular targets.

### 4.7. Virtual Screening of Molecular Targets of Vismione E

The pdb files of the IMPDH2 (PDB ID: 6I0M), MMP1 (PDB ID: 4AUO), ADAM17 (PDB ID: 2FV5), CDK8 CCNC (PDB ID: 4CRL) proteins were obtained from the RCSB Protein Data Bank (https://www.rcsb.org (accessed on 15 February 2023)) and prepared for docking using PrepDock of the UCFS Chimera 1.16 software. The chemical structures of ligands were prepared for docking using ChemOffice.

The docking was carried out on the SwissDock online server (http://www.swissdock.ch (accessed on 15 February 2023)) based on the docking software EADock DSS [55]. The predicted building modes for each target/ligand pair were visualized and analyzed using the UCFS Chimera 1.16 software.

Docking parameters such as Gibb’s free energy (ΔG, kcal/mol), full fitness (FF, kcal/mol), and hydrogen bonding (H-bond) were used for the analysis of target/ligand complexes.

### 4.8. Statistical Data Evaluation

All data were obtained in three independent replicates, and calculated values are expressed as a mean ± standard error mean (SEM). Student’s t-test was performed using SigmaPlot 14.0 (Systat Software Inc., San Jose, CA, USA) to determine statistical significance. The differences were considered statistically significant at *p* < 0.05.

## 5. Conclusions

The sponge-associated fungus *Aspergillus* sp. 1901NT-1.2.2 was found to be a source of the anthraquinone derivative vismione E, for which absolute stereochemistry data were established for the first time. Moreover, endocrocin was identified in the EtOAc extract of this fungus through the HPLC MS approach, and biosynthetic relationships between vismione E and endocrocin were proposed.

Vismione E showed significant cytotoxic effects against human breast cancer MCF-7 cells. It was found that vismione E can decrease MCF-7 cell viability, migration, and proliferation, as well as arrest the cell cycle in the G1 phase. It is likely that the anticancer activity of vismione E may be caused by its effects on cell proliferation machinery or nucleotide biosynthesis. The molecular docking of vismione E with predicted targets shows that inosine-5′-monophosphate dehydrogenase 2 (IMDH2) can be considered as one of the molecular targets for future investigation.

## Figures and Tables

**Table 1:** ^1^H and ^13^C NMR spectroscopic data (δ in ppm, 700 MHz, CDCl_3_) for **1**.

Pos.	δ_C_, Mult	δ_H_ (*J* in Hz)	HMBC
1	156.1, C		
2	114.8, C		
3	162.0, C		
4	97.8, CH	6.54, s	1 *, 2, 4a, 11 *, 9 *, 10
4a	108.1, C		
5	43.4, CH_2_	3.02, d (15.8) 3.06, d (15.8)	4a, 6, 7, 8a *, 10, 10a, 6-Me
6	71.0, C		
7	51.1, CH_2_	2.81, d (17.2) 2.85, d (17.2)	4a *, 5, 6, 6-Me, 8
8	201.5, C		
8a	139.0, C		
9	165.9, C		
9a	108.1, C		
10	117.6, CH	6.86, s	1 *, 4, 4, 5, 8, 8a, 9 *
10a	134.1, C		
11	22.0, CH_2_	3.44, d (7.1) 3.44, d (7.1)	1, 2, 3, 12, 13
12	122.3, CH	5.24, t (6.9)	2, 11, 14, 15
13	131.7, C		
14	17.8, CH_3_	1.81, s	12, 13, 15
15	25.8, CH_3_	1.68, s	12, 13, 14
1-OH	-	9.95, s	1, 2, 4a
3-OMe	55.6, CH_3_	3.92, s	3
6-Me	28.8, CH_3_	1.44, s	5, 6, 7, 8 *

*—weak interaction.

**Table 2:** The cytotoxic activity of vismione E (**1**).

PC-3	MCF-7	MCF-10A	H9c2
IC_50_, µM			
10.1 ± 2.1	9.0 ± 0.4	65.3 ± 2.2	69.3 ± 8.0

The data are presented as mean ± standard error of mean (SEM). All experiments were carried out in triplicate.

**Table 3:** Molecular docking data.

Target	ΔG (kcal/mol)	Full Fitness (kcal/mol)	Energy	H-Binding Residue/ H-Bonding Distance
Vismione E				
IMPDH2	−7.478466	−5339.8955	7.369	Lys229 H …O 2.165 Å
	−7.4588733	−5338.026	18.4965	Lys489 HN … O 2.678 Å H …O Glu487 3.265 Å
MMP1	−6.889332	−4766.2676	29.1129	H…O Gln974 2.119 Å
ADAM17	−7.782852	−3054.5269	12.3965	Lys455 H … O 3.173 Å Ala266 HN … O 2.051 Å H … O Ala270 2.679 Å Lys273 H … O 2.302 Å
CDK8CCNC	−7.634472	−3557.7717	15.7452	Lys18 H…O 2.056 Å
Mycophenolic acid				
IMPDH2	−7.7401905	−5380.3955	1.44649	Lys489 HN … O 2.830 Å Asp16 HN … O 2.136 Å H …O Pro14 1.805 Å
Theoretical de-prenylated derivative of vismione E				
IMPDH2	−6.7235613	−5343.706	22.4293	Lys489 HN … O 2.269 Å
	−6.6762652	−5344.5107	18.8873	H …O Asp15 2.911 Å

## Data Availability

Not applicable.

## Supplementary Materials

The following are available online at https://www.mdpi.com/article/10.3390/ijms24098150/s1.

## Appendix A

**Table A1:** The compounds identified in EtOAc extract of *Aspergillus* sp. 1901NT-1.2.2 strain.

N	Name	Structure	RT	Exact Mass (Measured)	Exact Mass (Calcd)	Δ, ppm	MQScore (GNPS)	Ref.
1		C_9_H_8_O_5_	3.59	195.0288 [M + H]^+^	195.0288	0		
2	7-hydroxy-3-(2-hydroxypropyl)-5-methylisochromen-1-one		5.12	235.0970 [M + H]^+^	235.0965	−2.2	0.89	[19]
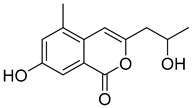
3		C_11_H_10_O_3_	6.18	191.0709 [M + H]^+^	191.0703	−3.3		
4		C_17_H_14_O_6_	6.96	315.0870 [M + H]^+^	315.0863	−2.2		
5	endocrocin		8.16	315.0504 [M + H]^+^	315.0499	−1.5	0.93	[20]
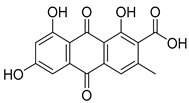
6		C_15_H_12_O_5_	8.47	273.0745 [M + H]^+^	273.0758	4.6		
7		C_21_H_24_O_6_	9.08	373.1643 [M + H]^+^	373.1646	0.7		
8		C_21_H_24_O_6_	9.60	373.1643 [M + H]^+^	373.1646	0.7		
9		C_20_H_26_O_4_	10.95	331.1906 [M + H]^+^	331.1904	−0.6		
10		C_30_H_49_NO_11_	11.16	600.3391 [M + H]^+^	600.3378	−2.1		
11		C_20_H_22_O_5_	12.16	343.1542 [M + H]^+^	343.1540	−0.6		
12	11a-hydroxy-4,4,9-trimethyl-9-vinyl-1,2,3,4,9,10,11,11a-octahydrodibenzo[c,e]oxepine-5,7-dione		13.16	317.1750 [M + H]^+^	317.1747	−0.8	0.83	[56]
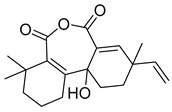
13		C_18_H_19_N_2_O_8_	13.26	391.1129 [M + H]^+^	391.1136	1.8		
14		C_21_H_20_O_6_	13.75	369.1306 [M + H]^+^	369.1333	7.2		
15		C_20_H_28_O_3_	14.28	317.2115 [M + H]^+^	317.2111	−1.2		
16	vismione E		15.59	357.1700 [M + H]^+^	357.1697	0.2		[21,22,23,24]
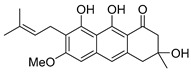
17		C_20_H_20_O_9_	16.05	427.0996 [M + Na]^+^	427.0999	0.8		
18		C_26_H_22_O_6_	16.57	453.1299 [M + Na]^+^	453.1309	2.1		
19		C_17_H_14_O_4_	17.67	283.0975 [M + H]^+^	283.0965	−3.6		
20		C_21_H_36_O_4_	18.39	353.2657 [M + H]^+^	283.2686	8.3		
21		C_20_H_30_O	18.75	287.2372 [M + H]^+^	287.2369	−0.9		
22		C_30_H_38_O_4_	19.15	463.2826 [M + H]^+^	463.2843	3.6		
23		C_24_H_38_O_4_	20.35	413.2674 [M + Na]^+^	413.2662	−2.8		

## Appendix B

**Table A2:** The top 50 predicted molecular targets of vismione E based on SwissTargetPrediction.

Target	Common Name	Uniprot ID	ChEMBL ID	Probability
Inosine-5′-monophosphate dehydrogenase 2	IMPDH2	P12268	CHEMBL2002	0.109339753231
Matrix metalloproteinase 1	MMP1	P03956	CHEMBL332	0.109339753231
ADAM17	ADAM17	P78536	CHEMBL3706	0.109339753231
CDK8/Cyclin C	CCNC CDK8	P24863 P49336	CHEMBL3038474	0.109339753231
Cell division protein kinase 8	CDK8	P49336	CHEMBL5719	0.109339753231
PI3-kinase p110- alpha subunit	PIK3CA	P42336	CHEMBL4005	0.109339753231
Proto-oncogene tyrosine-protein kinase ROS	ROS1	P08922	CHEMBL5568	0.109339753231
Interleukin-8 receptor B	CXCR2	P25025	CHEMBL2434	0.109339753231
Tyrosine-protein kinase SYK	SYK	P43405	CHEMBL2599	0.109339753231
Leucine-rich repeat serine/threonineprotein kinase 2	LRRK2	Q5S007	CHEMBL1075104	0.109339753231
Cyclin-dependent kinase 1/cyclin B	CCNB3 CDK1 CCNB1 CCNB2	Q8WWL7 P06493 P14635 O95067	CHEMBL2094127	0.109339753231
Mammalian target of rapamycin (mTORC1)	FKBP1A MTOR	P62942 P42345	CHEMBL2221341	0.109339753231
Phosphodiesterase 5A	PDE5A	O76074	CHEMBL1827	0.109339753231
Matrix metalloproteinase 9	MMP9	P14780	CHEMBL321	0.109339753231
Interleukin-1 receptor-associated kinase 4	IRAK4	Q9NWZ3	CHEMBL3778	0.109339753231
Corticotropin releasing factor receptor 1	CRHR1	P34998	CHEMBL1800	0.109339753231
Matrix metalloproteinase 3	MMP3	P08254	CHEMBL283	0.109339753231
Thrombin and coagulation factor X	F10	P00742	CHEMBL244	0.109339753231
Protein farnesyltransferase	FNTA FNTB	P49354 P49356	CHEMBL2094108	0.109339753231
Phosphodiesterase 2A	PDE2A	O00408	CHEMBL2652	0.109339753231
P2X purinoceptor 3	P2RX3	P56373	CHEMBL2998	0.109339753231
Macrophage colony stimulating factor receptor	CSF1R	P07333	CHEMBL1844	0.109339753231
Tyrosine-protein kinase JAK3	JAK3	P52333	CHEMBL2148	0.109339753231
Protein kinase C gamma	PRKCG	P05129	CHEMBL2938	0.109339753231
Protein kinase C delta	PRKCD	Q05655	CHEMBL2996	0.109339753231
Protein kinase C alpha	PRKCA	P17252	CHEMBL299	0.109339753231
Cyclin-dependent kinase 4/cyclin D1	CCND1 CDK4	P24385 P11802	CHEMBL1907601	0.109339753231
Cyclin-dependent kinase 2/cyclin E	CCNE2 CDK2 CCNE1	O96020 P24941 P24864	CHEMBL2094126	0.109339753231
Casein kinase II alpha	CSNK2A1	P68400	CHEMBL3629	0.109339753231
Cyclin-dependent kinase 2/cyclin A	CDK2 CCNA1 CCNA2	P24941 P78396 P20248	CHEMBL2094128	0.109339753231
PI3-kinase p110- beta subunit	PIK3CB	P42338	CHEMBL3145	0.109339753231
Nerve growth factor receptor Trk-A	NTRK1	P04629	CHEMBL2815	0.109339753231
p53-binding protein Mdm-2	MDM2	Q00987	CHEMBL5023	0.109339753231
Serine/threonineprotein kinase SMG1	SMG1	Q96Q15	CHEMBL1795195	0.109339753231
Tyrosine-protein kinase ABL	ABL1	P00519	CHEMBL1862	0.109339753231
Proto-oncogene vav	VAV1	P15498	CHEMBL3259472	0.109339753231
Phosphodiesterase 10A	PDE10A	Q9Y233	CHEMBL4409	0.109339753231
