# Supplementary material for: Absolute Stereochemistry and Cytotoxic Effects of Vismione E from Marine Sponge-Derived Fungus Aspergillus sp. 1901NT-1.2.2
Source: Int J Mol Sci. 2023 May 2;24(9):8150. doi: 10.3390/ijms24098150 (PMC10179051; doi:10.3390/ijms24098150)

Supplementary Materials

Anthraquinone vismione E from marine sponge-derived fungus *Aspergillus* sp. 1901NT-1.2.2 and its cytotoxic activity

Elena V. Girich[1,†], Phan Thi Hoai Trinh[2,†], Dmitry V. Berdyshev[1,†], Liliana E. Nesterenko[1,3], Roman S. Popov[1], Natalya Yu. Kim[1], Anton B. Rasin[1], Ekaterina S. Menchinskaya[1], Aleksandra S. Kuzmich[1], Ekaterina A. Chingizova[1], Artem S. Minin[4, 5], Ngo Thi Duy Ngoc[2], Tran Thi Thanh Van[2], Ekaterina A. Yurchenko[1,*], and Anton N. Yurchenko[1,]

Abstract: The metabolic profile of *Aspergillus* sp. 1901NT-1.2.2 sponge-associated fungal strain was investigated by HPLC MS technique. Only two minor peaks were identified with endocrocin and terpene derivative MS data from GNPS database. Main compound was isolated and identified with known anthraquinone derivative vismione E. Absolute stereochemistry of vismione E was established for the first time by ECD and quantum chemical methods. Vismione E shown high cytotoxic activity against human breast cancer MCF-7 cells with IC_{50} of 9.0 μM in compare with low toxicity for normal human breast MCF-10A cells with IC_{50} of 65.3 μM. Using three independent techniques, it was found that vismione E inhibits MCF-7 cell proliferation and arrest cell cycle in G1 phase. Moreover, negative influence of vismione E on MCF-7 cell migration was detected. . Molecular docking of vismione E suggested the IMPDH2 enzyme as one of the molecular targets for this anthraquinone derivative.

Keywords: *Aspergillus*; marine-derived fungus; vismione E; HPLC MS; secondary metabolites; cytotoxicity; MCF-7; proliferation.

Figure S1. ^{1}H NMR spectrum of vismione E (1) 3
Figure S2. ^{13}C NMR spectrum of vismione E (1) 4
Figure S3. DEPT ^{13}C NMR spectrum of vismione E (1) 5
Figure S4. HSQC spectrum of vismione E (1) 6
Figure S5. HMBC spectrum of vismione E (1) 7
Figure S6. ^{1}H-^{1}H COSY spectrum of vismione E (1) 8
Figure S7. ROESY spectrum of vismione E (1) 9
Figure S8. UV spectrum of vismione E (1) 10
Figure S9. CD spectrum of vismione E (1) 10
Figure S10. HR (–)ESI MS spectrum of vismione E (1) 11
Figure S11. HR (+)ESI MS spectrum of vismione E (1) 11
Figure S12. Photo of silicon inserts for 24-well plates, used for investigation of cell migration 12
S13. Description of quantum-chemical modeling 12
Table S14. The optimal values for the UV shifts 12
S15. Conformation analysis 13
Figure S16. The scan of potential energy surface along the IRC trajectory, calculated for the inversion of ring C 14
Figure S17. Theoretical dependencies of dihedral angles, characterizing the distortion of ring C during the EQ$\rightarrow$TS1$\rightarrow$AX inversion process 14
Figure S18. The scan of potential energy surface along the dihedral angle θ_{LAM4}) 15
Figure S19. The variation of the potential energy along LAM2 coordinate 15
Figure S20. The most stable conformations of 6*S*-1 16
Figure S21. ECD spectra for main conformations of 6*S*-1 17
Figure S22. Contribution of main conformations to total ECD spectrum of 6*S*-1 17
Figure S23. MS/MS spectra of HPLC MS peak #5 (endocrocin) 18
Figure S24. MS/MS spectra of HPLC MS peak #12 (11a-hydroxy-4,4,9-trimethyl-9-vinyl-1,2,3,4,9,10,11,11a-octahydrodibenzo[c,e]oxepine-5,7-dione) 18

Figure S1. ^{1}H NMR spectrum of vismione E (1)

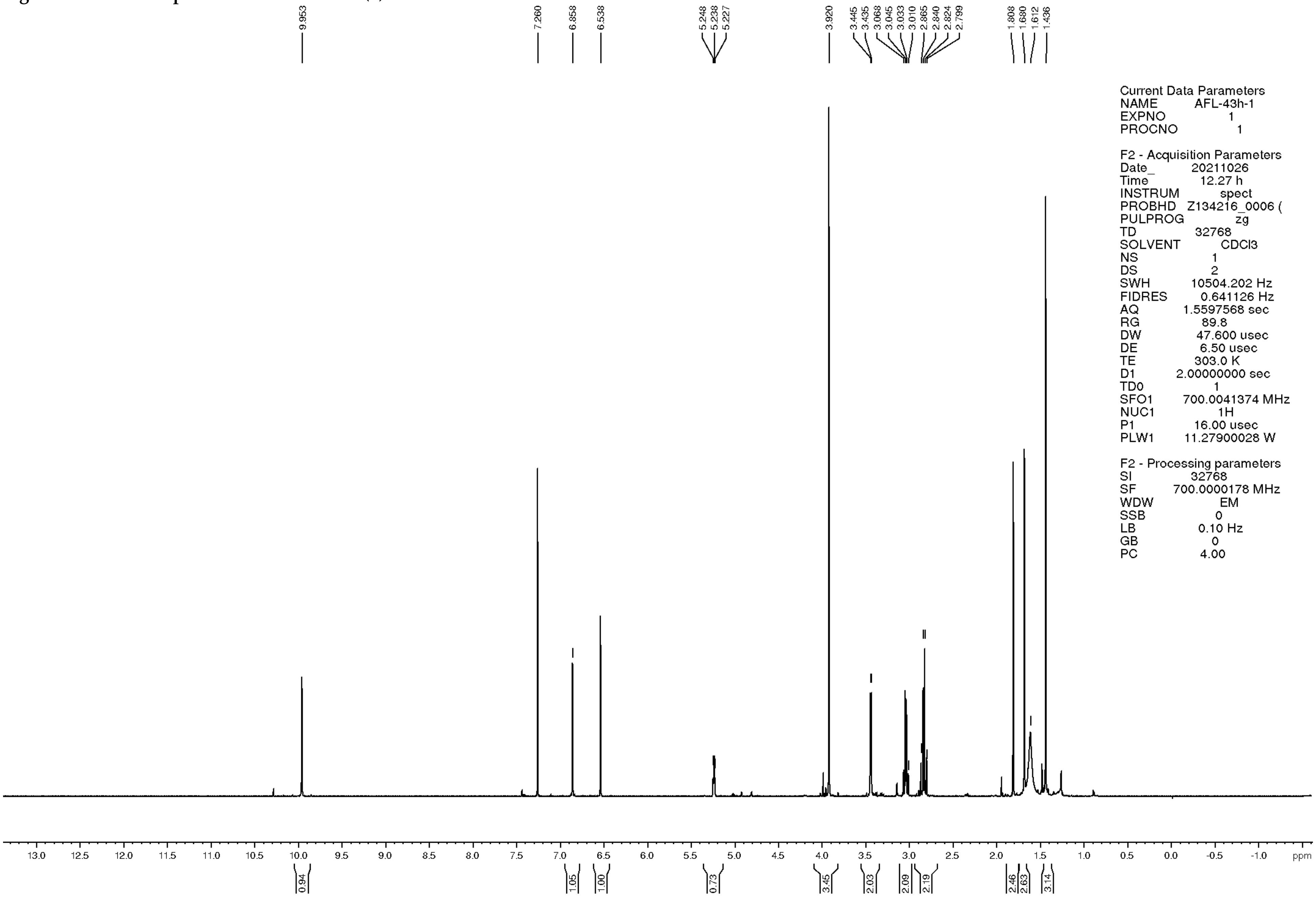

Figure S2. ^{13}C NMR spectrum of vismione E (1)

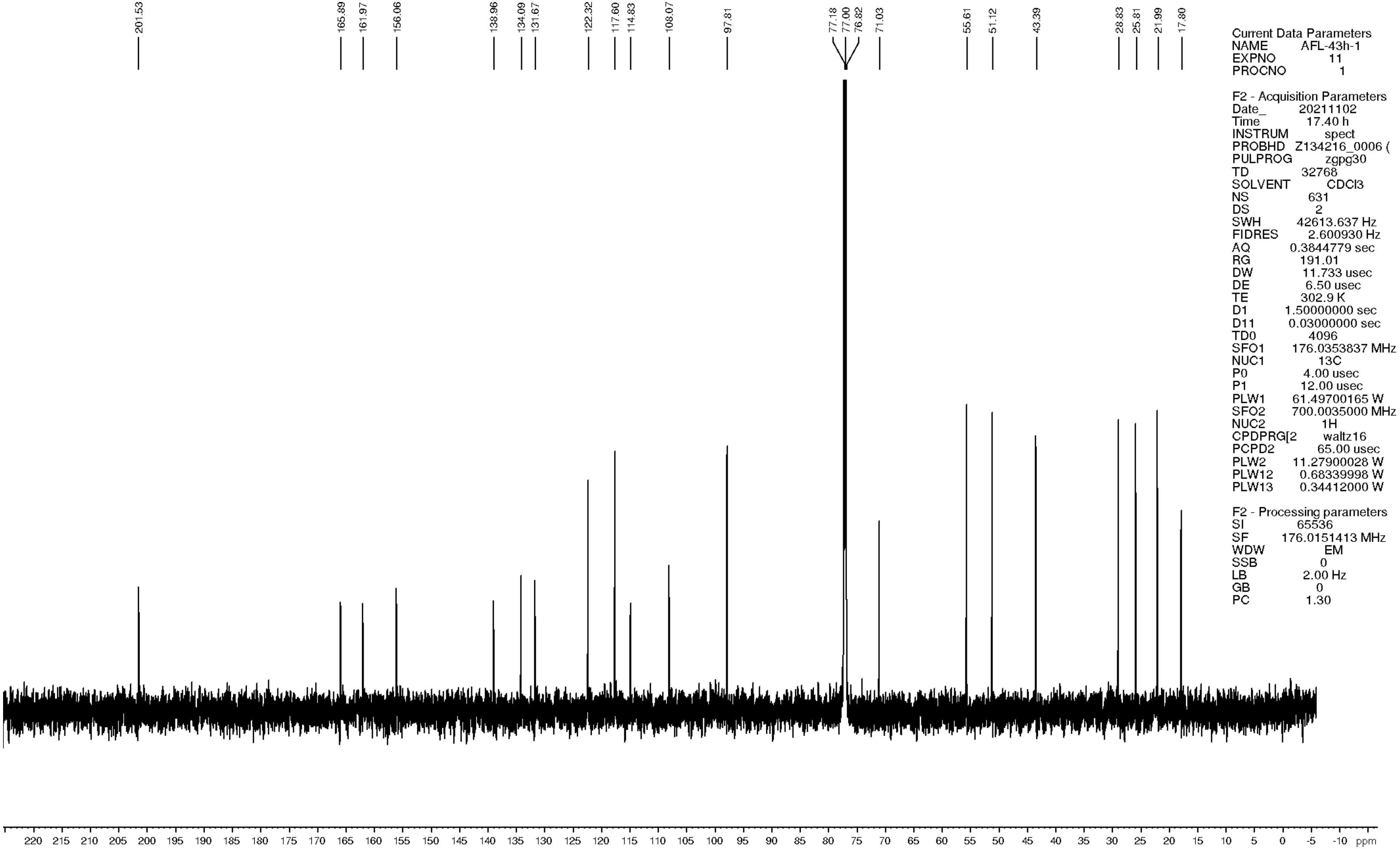

Figure S3. DEPT ^{13}C NMR spectrum of vismione E (1)

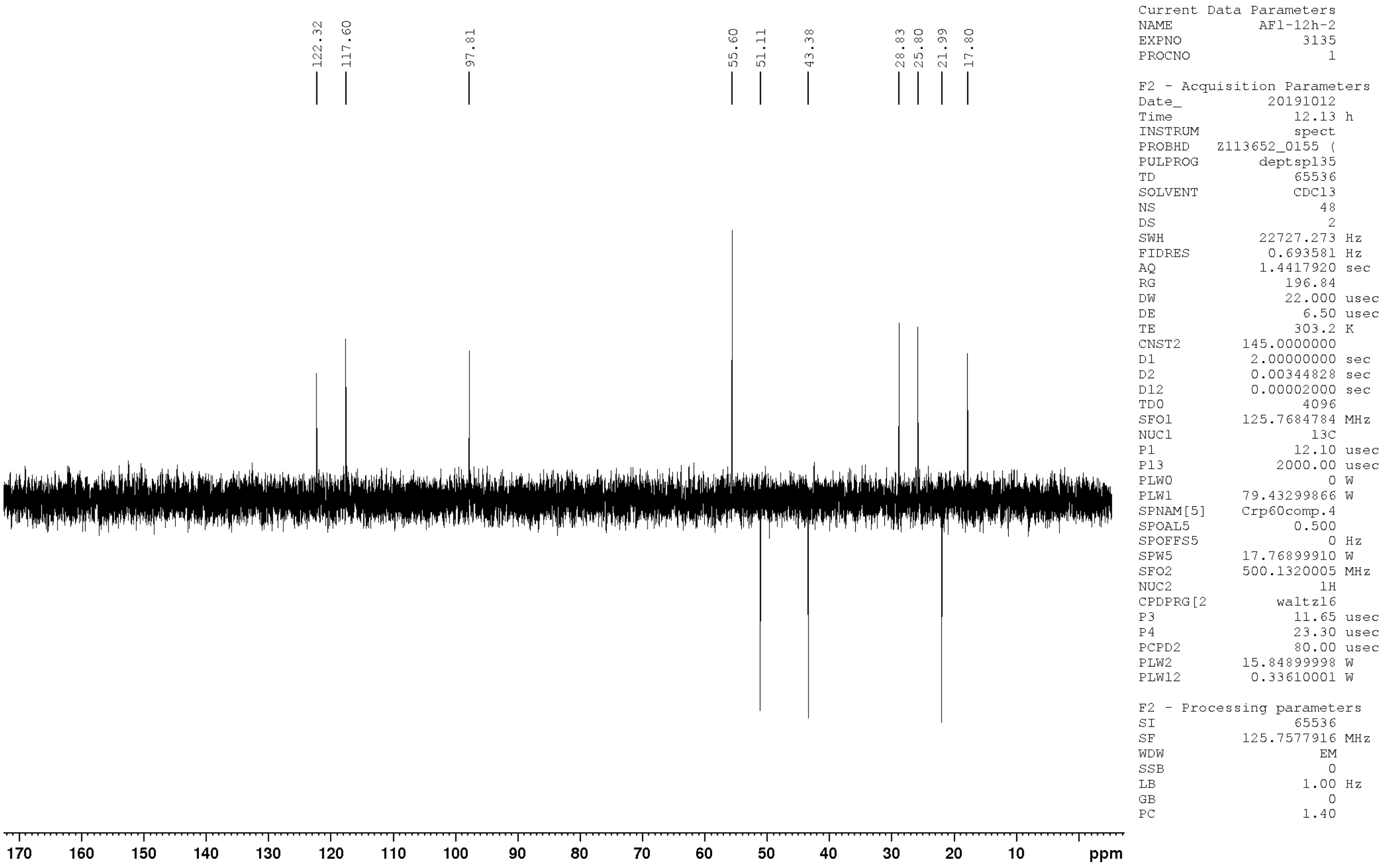

Figure S4. HSQC spectrum of vismione E (1)

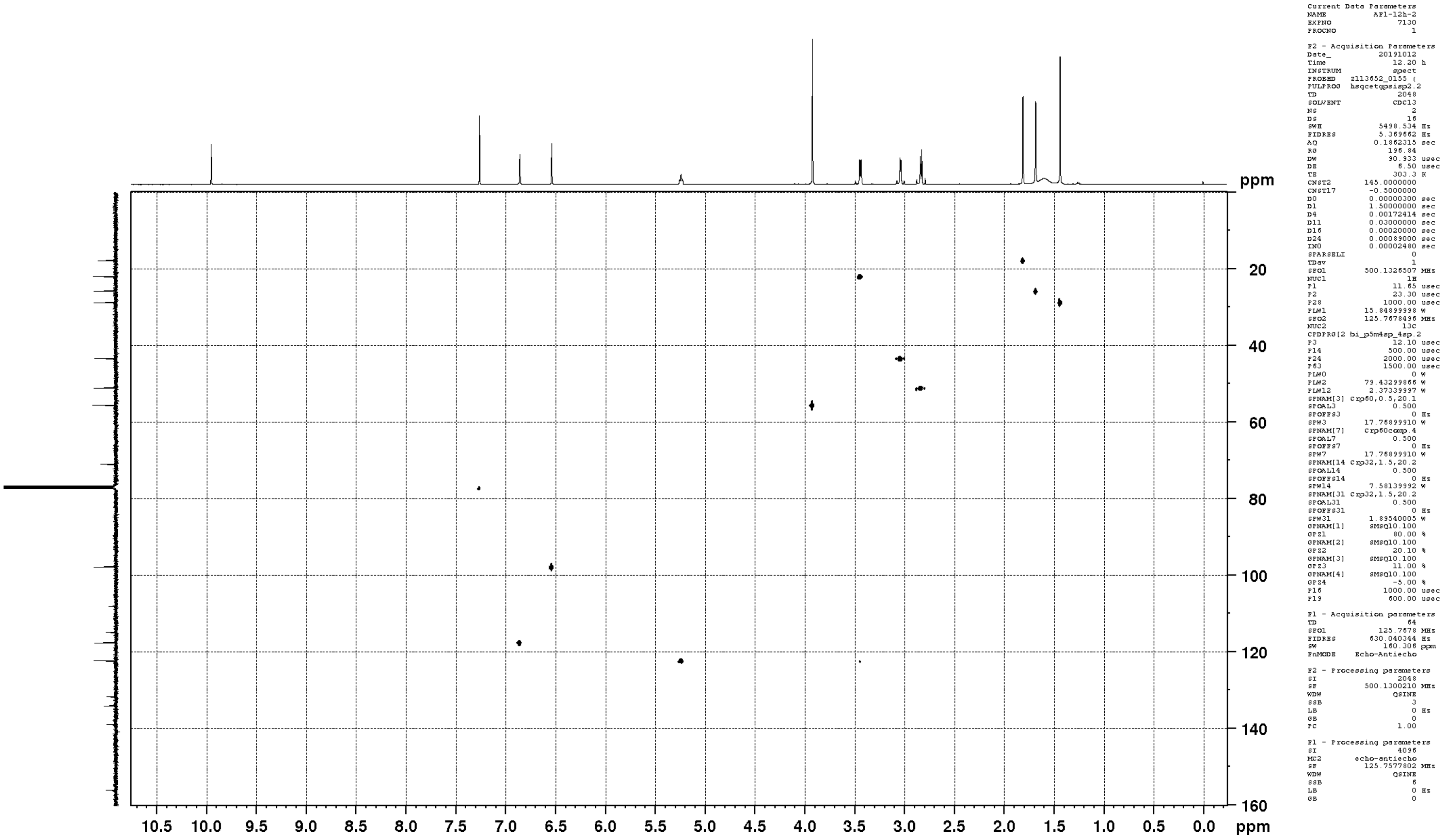

Figure S5. HMBC spectrum of vismione E (1)

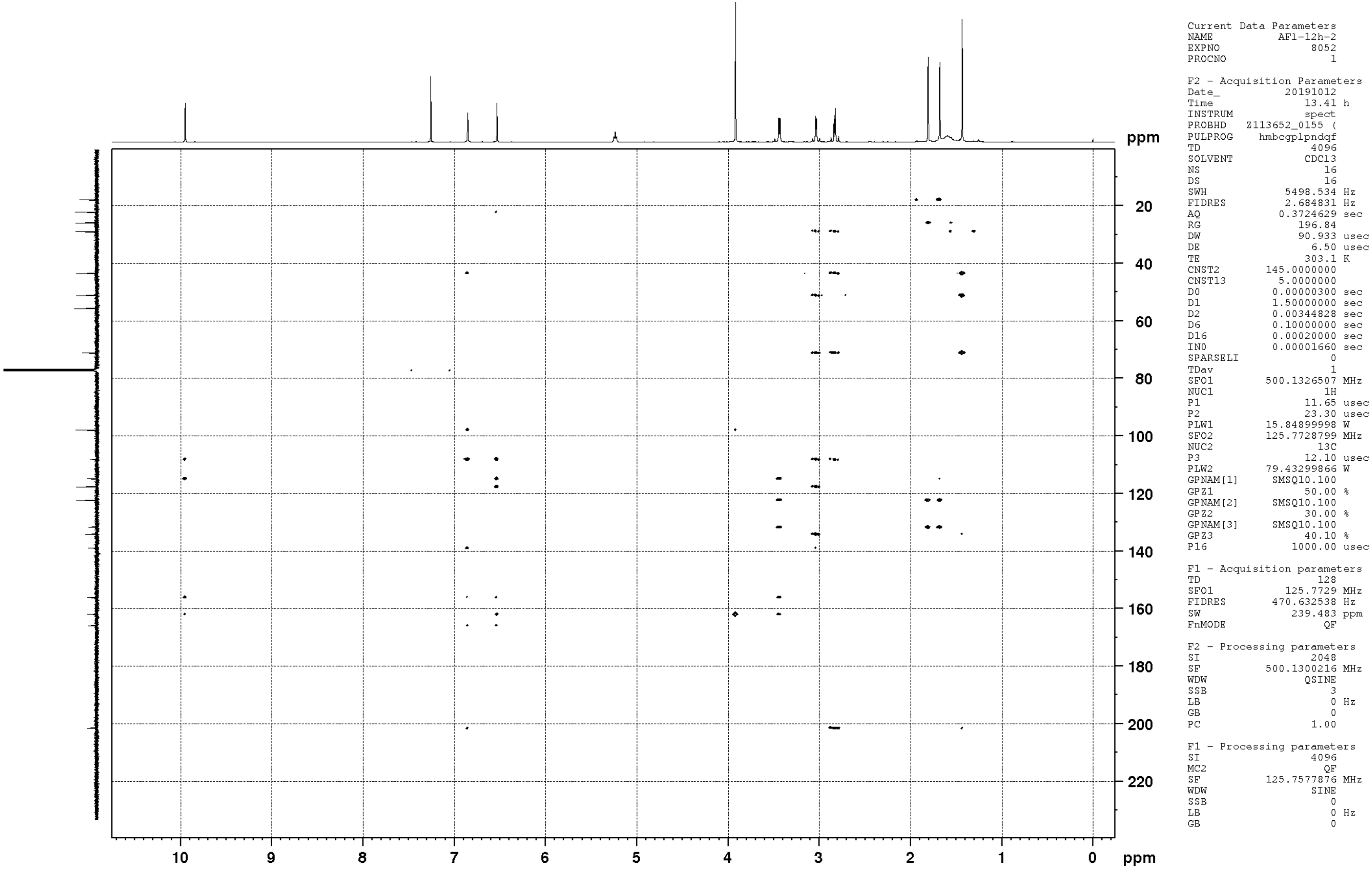

Figure S6. ¹H-¹H COSY spectrum of vismione E (1)

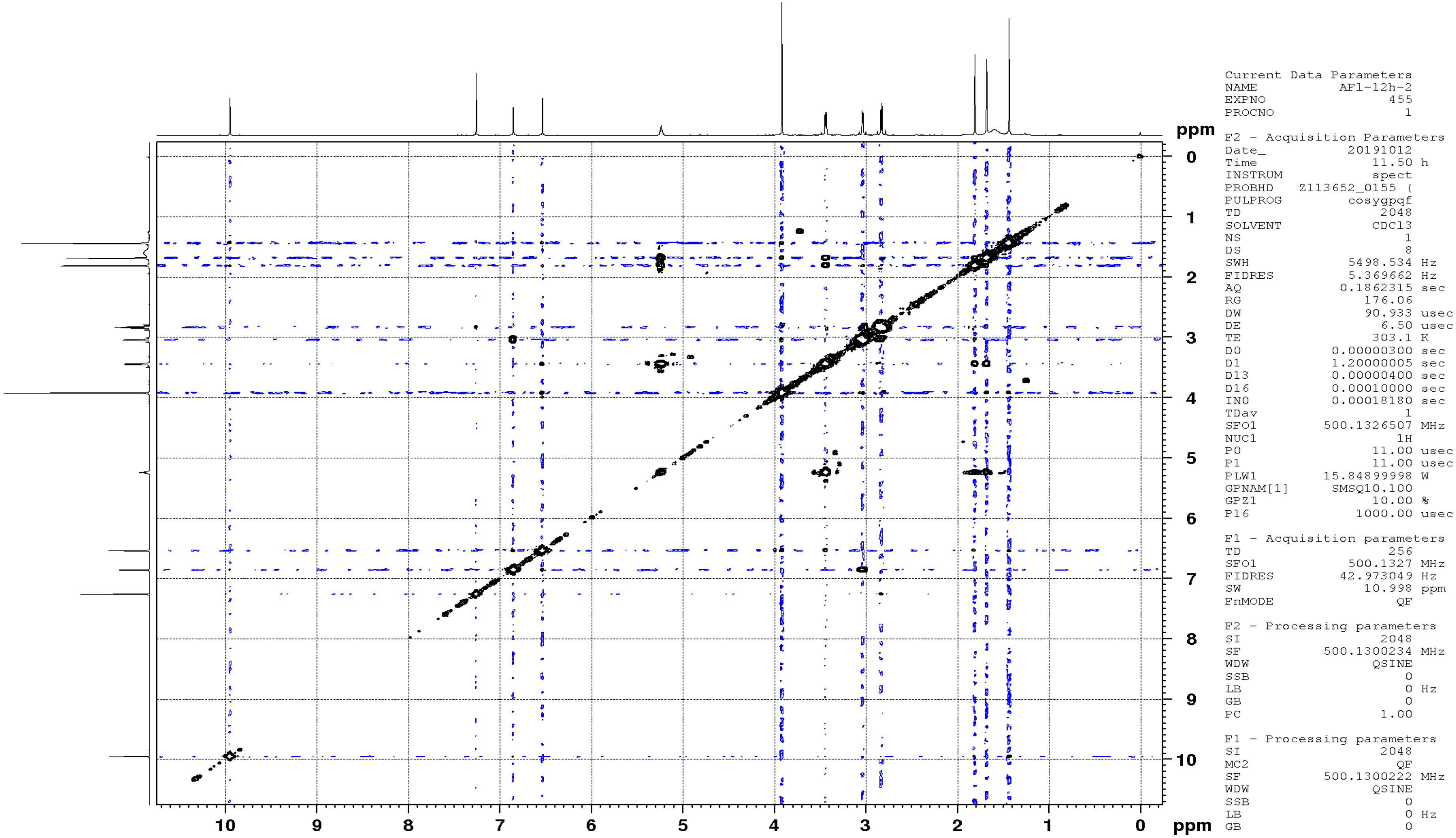

Figure S7. ROESY spectrum of vismione E (1)

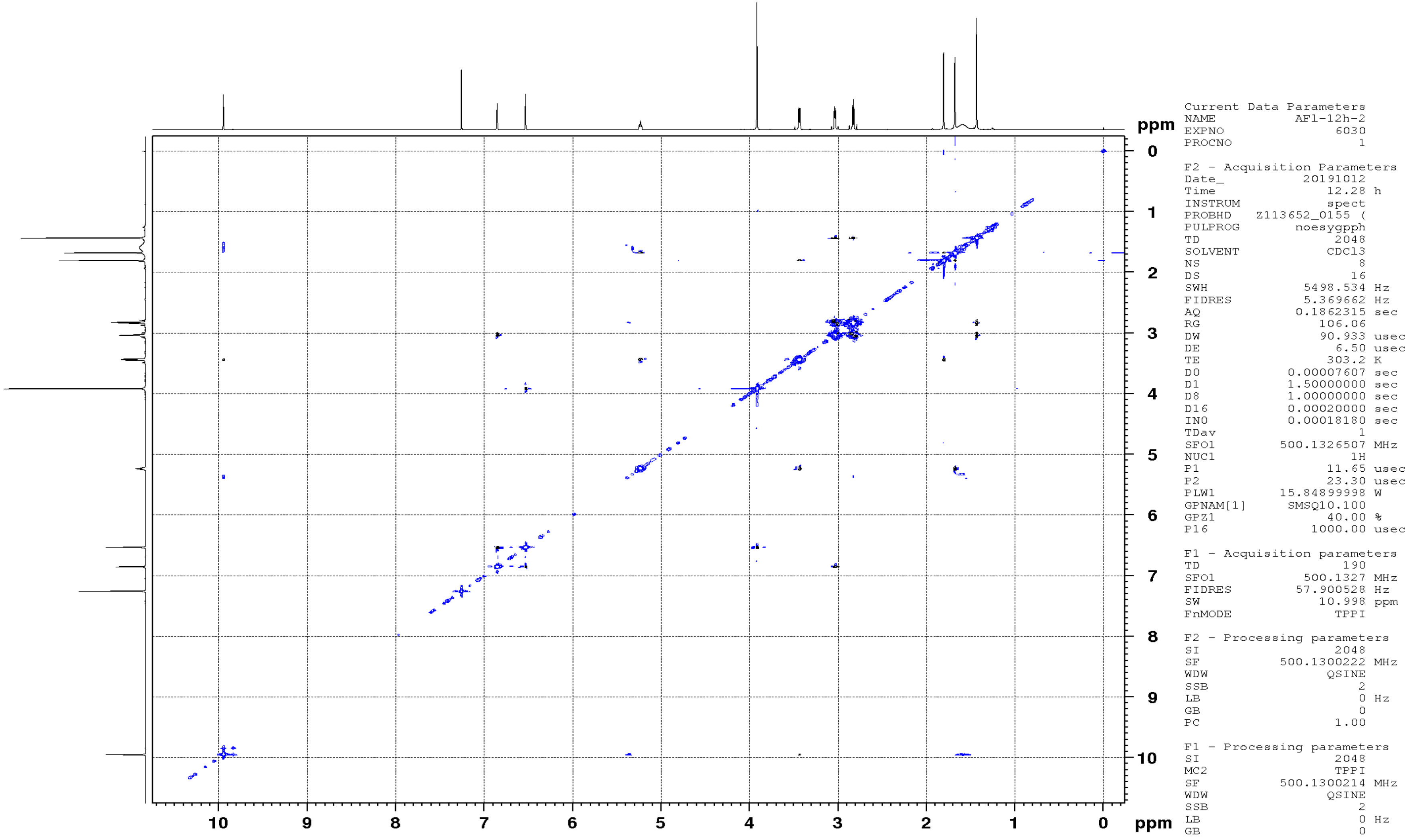

Figure S8. UV spectrum of vismione E (1)

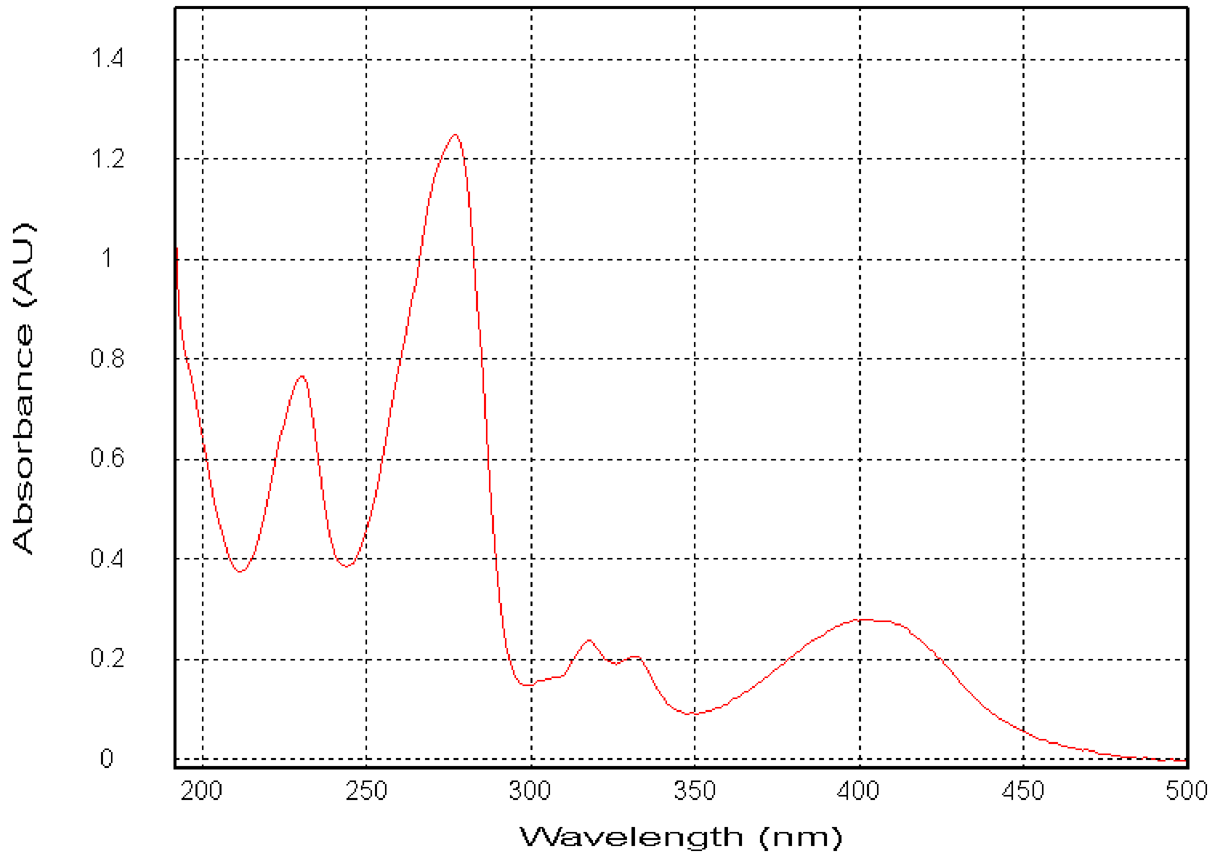

Figure S9. CD spectrum of vismione E (1)

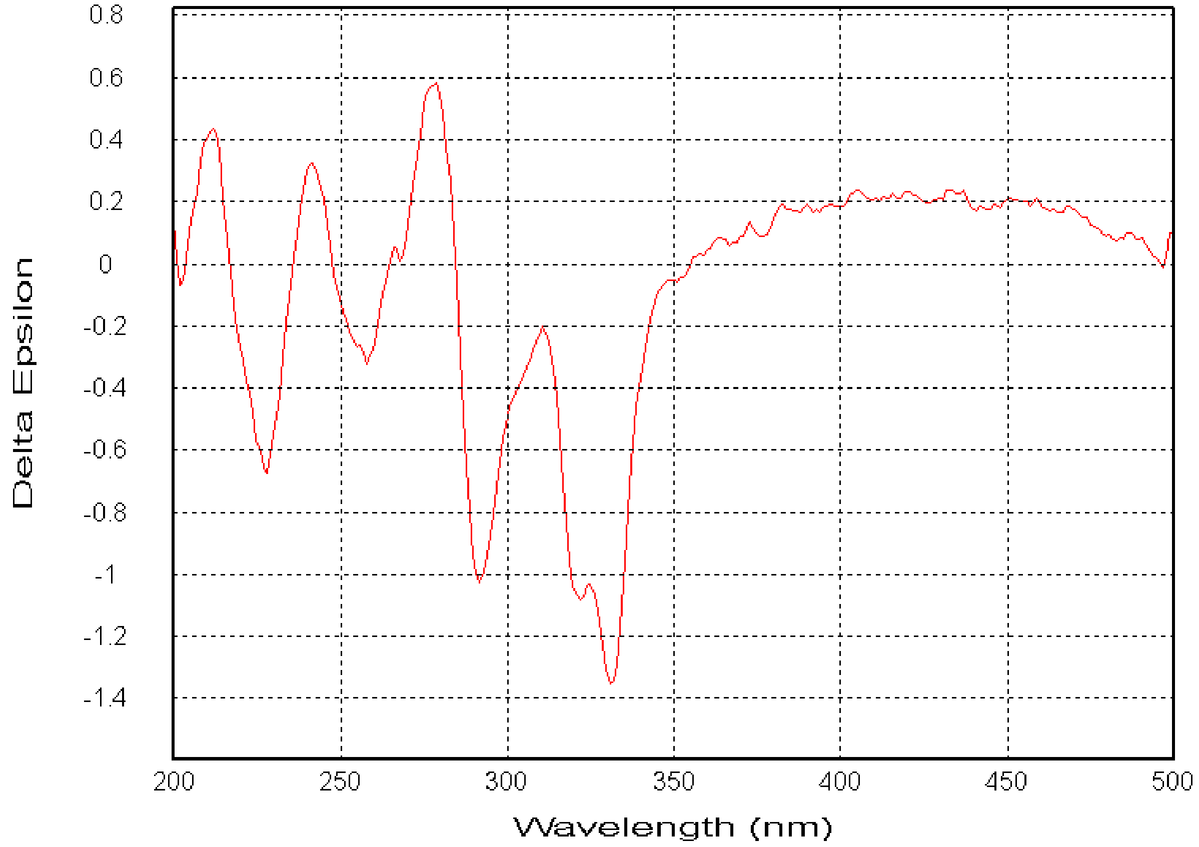

Figure S10. HR (–)ESI MS spectrum of vismione E (1)

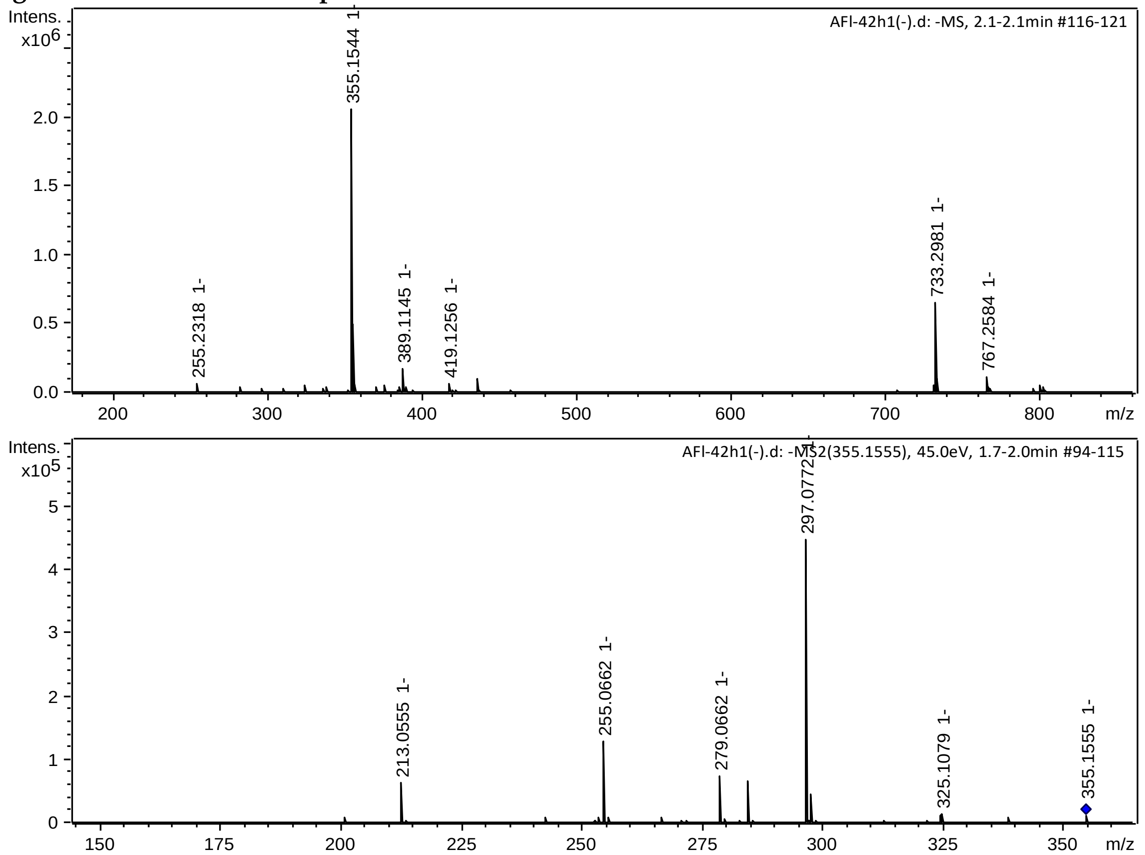

Figure S11. HR (+)ESI MS spectrum of vismione E (1)

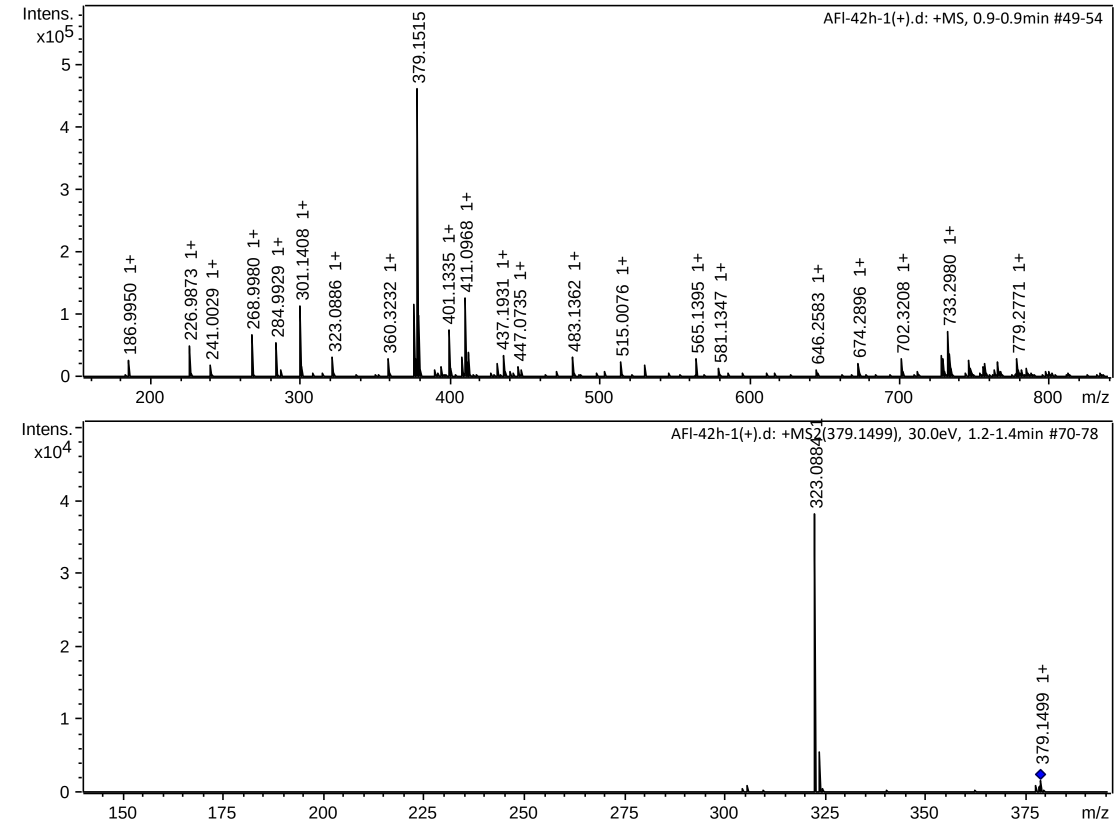

Figure S12. Photo of silicon inserts for 24-well plates, used for investigation of cell migration

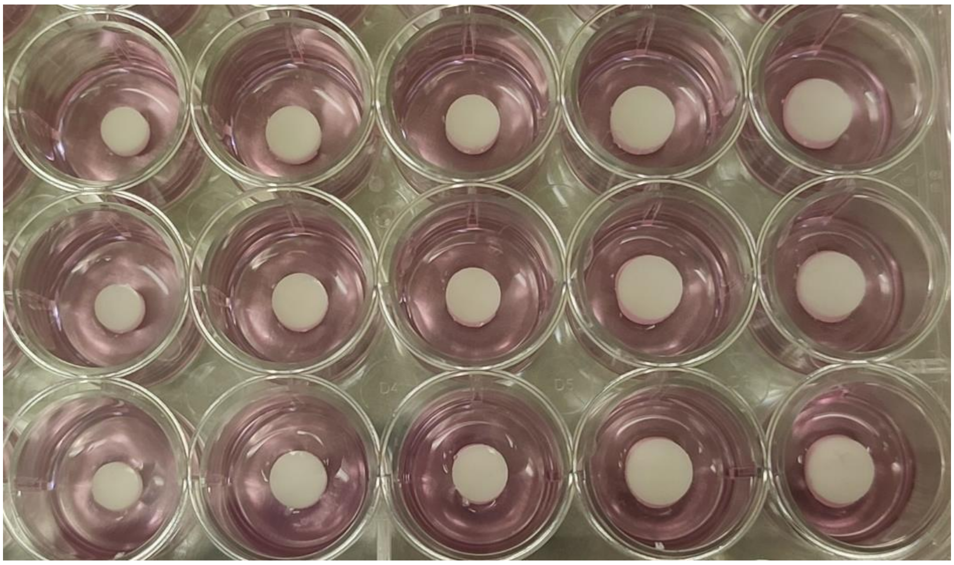

S13. Description of quantum-chemical modeling

The quantum-chemical calculations for 1 in CH3OH solvent were done using density functional theory (DFT) and the polarization continuum model (PCM), implemented in the Gaussian 16 package of programs. Different exchange-correlation functionals were used for calculation of thermodynamic and spectral properties of molecule 1: B3LYP, B3PW91, cam-B3LYP, LC-wPBE, PBE1PBE, BRxBRc and HISSaPBE.

First, the conformational analysis was done with B3LYP/6-311G(d), B3LYP/cc-pvTz and B3PW91/6-311++G(d) methods and the statistical weights (gim) of different conformations were obtained according to equation:

$$g_{im} = e^{-\Delta G_{im}/RT} \Big/ \sum_i e^{-\Delta G_{im}/RT} \qquad (1)$$

where the summation was done over eight most stable conformations of 1; the subscript "m" denotes conformation, for which G is minimal.

The excitation energies and the rotatory strengths were calculated using time-dependent density functional theory (TDDFT). Each individual transition from electronic ground state to the i-th calculated excited electronic state ($1 \le i \le 35$) was simulated as a Gauss-type function. The same value $\zeta = 0.16$ eV for the bandwidths at 1/e peak heights was used.

The total theoretical UV and ECD spectra were obtained after statistical averaging over all selected conformations:

$$Absorbance_{calc}(\lambda) = \sum_i g_i \cdot Absorbance_{i,calc}(\lambda) \qquad (2)$$

$$\Delta\varepsilon_{calc}(\lambda) = \sum_i g_i \cdot \Delta\varepsilon_{i,calc}(\lambda) \qquad (3)$$

The scaled theoretical and experimental spectra were obtained according to equations:

$$F_{scaled}(\lambda) = F(\lambda) \Big/ \left|F(\lambda_{peak})\right| \qquad (4)$$

,

where F is Absorbance or $\Delta\varepsilon$ and the denominator $|F(\lambda peak)|$ is a modulo of the peak value for the chosen characteristic band in corresponding spectrum.

The used density functionals differs in their ability to reproduce UV and ECD spectra. This realizes that the positions of bands in theoretical UV spectra are shifted relative to positions of corresponding bands in experimental spectrum. To improve the correspondence between calculated and experimental UV spectra we used the UV shifts $\Delta\lambda$ (one and the same for all bands in UV spectrum, calculated with taken functional, Table S14).

Table S14. The optimal values for the UV shifts

Functional	Δλ/nm	functional	Δλ/nm
B3LYP	0	Cam-B3LYP	+26
B3PW91	0	LC-wPBE	+40
PBE1PBE	+27	BRxBRc	-24
HISSaPBE	+37		

S15. Conformation analysis

Compound **1** is very flexible. Five Large-Amplitude Motions (LAM) along with the tautomeric rearrangement may proceed in this compound at one and the same time:

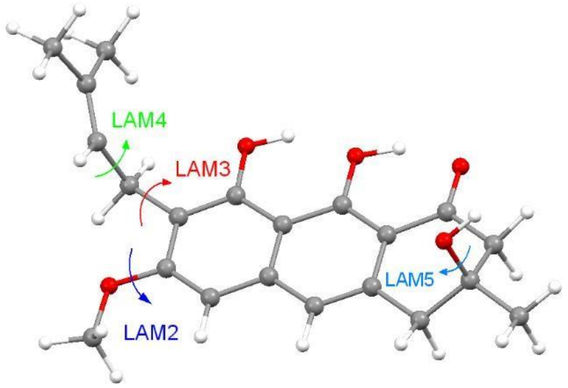

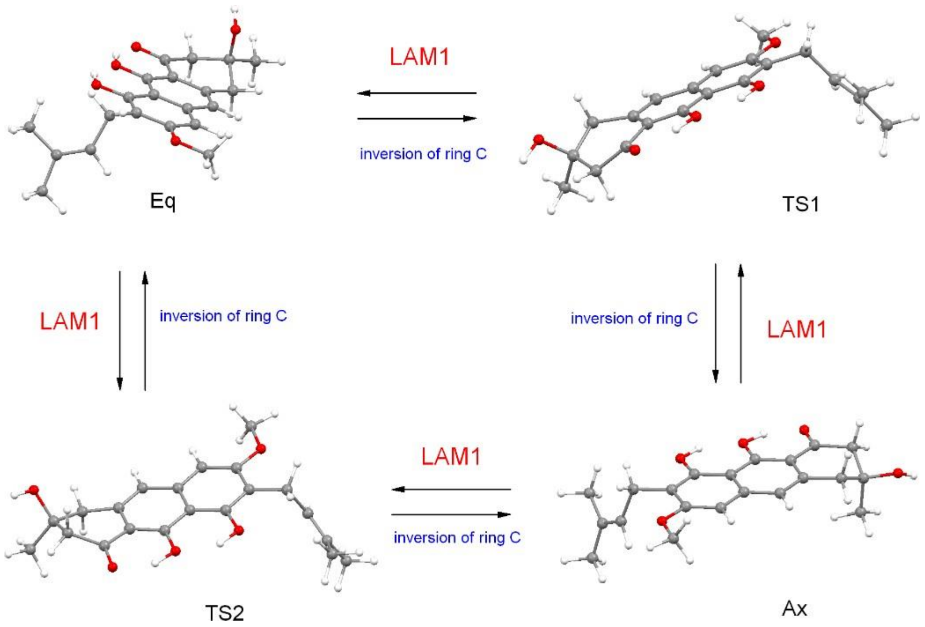

To distinguish different conformations the notation was used:

Conformation ≡αβγ, where index "α" denotes "AX" or "EQ" conformation of ring C; indexes "β" and "γ" determines the values of dihedral angles θLAM3 and θLAM4 correspondingly:

$\beta \equiv$ "a" when $0° \le \theta LAM3 \le +180°$; $\beta \equiv$ "b" when $180° \le \theta LAM3 \le 360°$;

$\gamma \equiv$ "a" when $+90° \le \theta LAM4 \le +180°$; $\gamma \equiv$ "b" when $-90° \le \theta LAM3 \le -180°$;

$\theta LAM3 \equiv \angle$ C12–C11–C2–C1; $\theta LAM4 \equiv \angle$C13–C12–C11–C2

$\theta LAM2 \equiv \angle$Me–O–C3–C2

Figure S16. The scan of potential energy surface along the IRC trajectory, calculated for the inversion of ring C

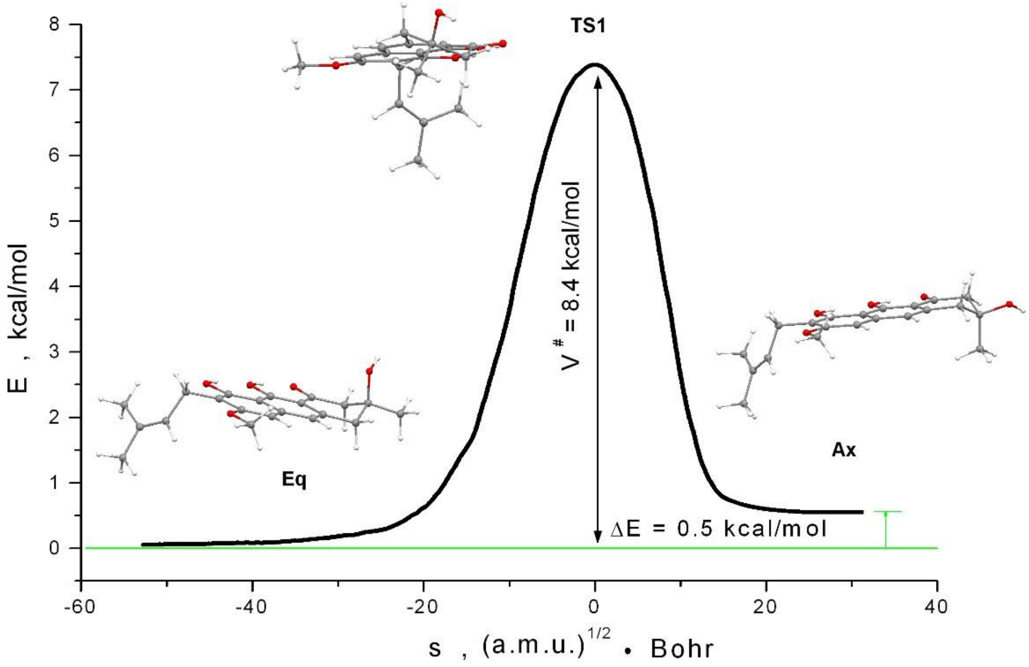

Figure S17. Theoretical dependencies of dihedral angles, characterizing the distortion of ring C during the EQ→TS1→AX inversion process

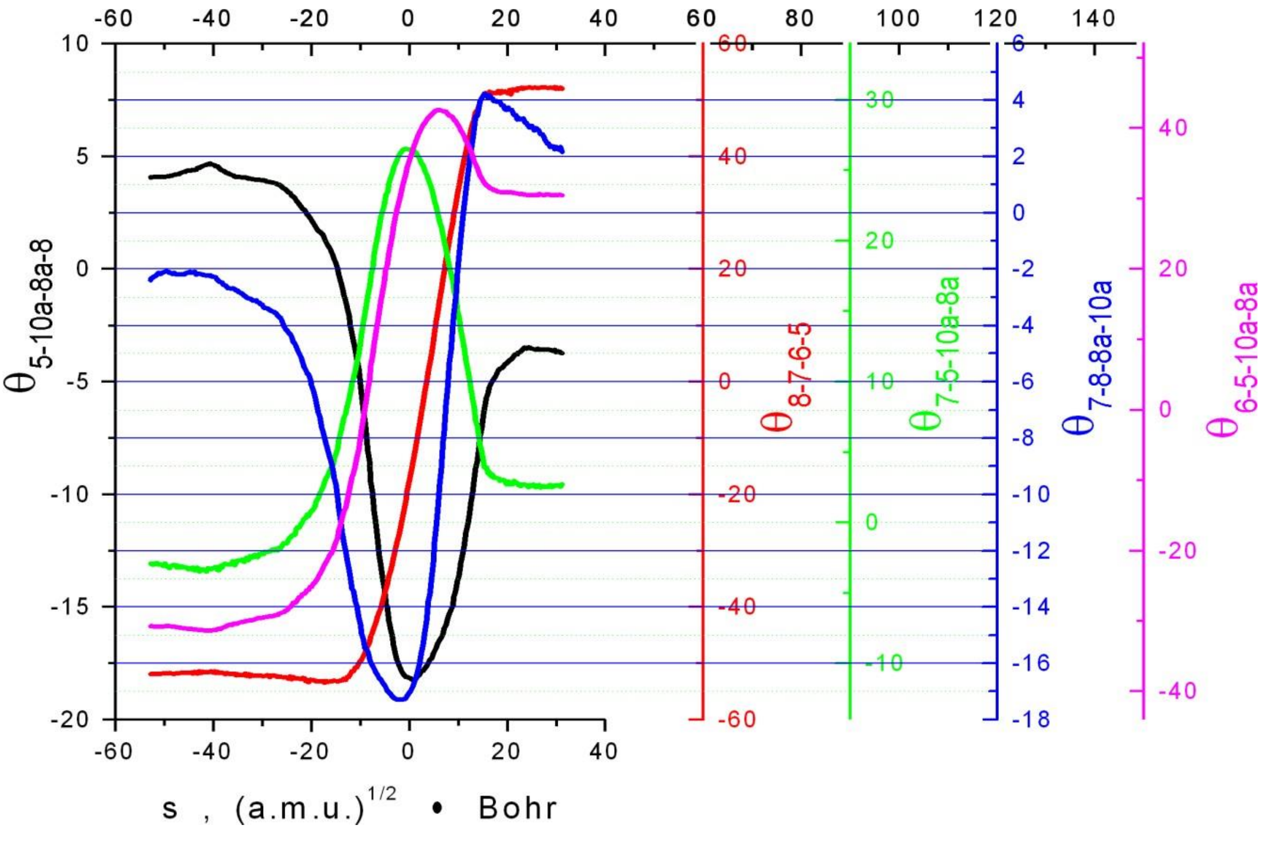

Figure S18. The scan of potential energy surface along the dihedral angle θ_{LAM4})

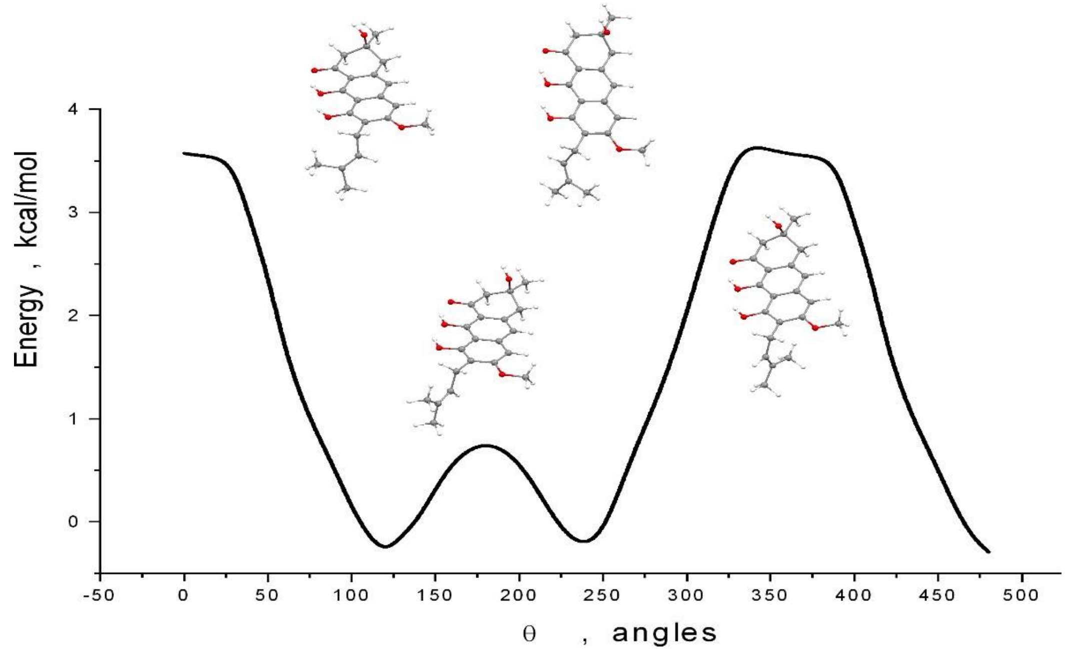

The internal rotation of the 3-OMe group around C–C bond is more constrained, but for -120° ≤ θLAM2 ≤ -240° the total energy of molecule varies in small diapason ΔE ≤ Emin + 4 kcal/mol

Figure S19. The variation of the potential energy along LAM2 coordinate

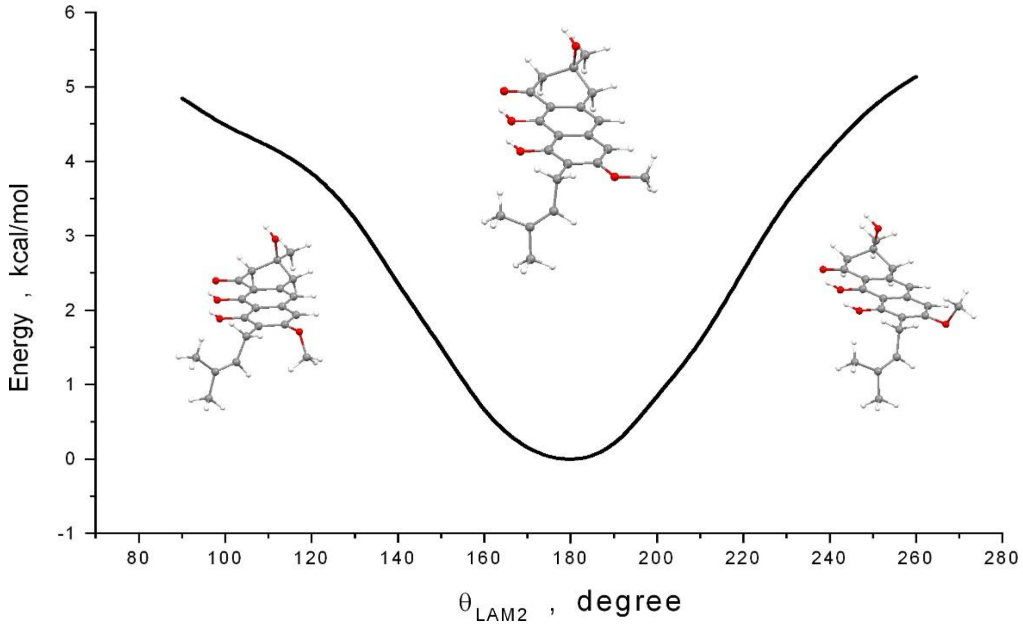

The obtained dependencies point out, that there are many distorted conformations of 1, which may contribute appreciably to the experimentally recorded spectra (at least these are conformations with 150° ≤ θLAM2 ≤ 210° and 60° ≤ θLAM4 ≤ 190°). The inversion of ring C may proceed via overpassing through two different transition states; the heights of potential energy barriers for these two reaction paths are:

ΔV#(EQ→TS1) ≈ 7.4 kcal/mol; ΔV#(EQ→TS2) ≈ 7.6 kcal/mol

The evolution of **1**'s geometry during the motion near potential energy minimums (denoted as "EQ" for equatorial and "AX" for axial orientations of Me subtituent at C-6) is complicated (Supplementary materials, Figure S17.). For energies E ≤ Emin + 1.5 kcal/mol many dihedral angles have values, which deviate significantly from their equilibrium values. Thus, the averaging over the inversion process can also noticeably contribute to the detecting spectral data.

Figure S20. The most stable conformations of 6*S*-1

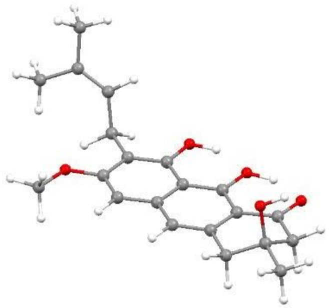<br>Sbb_Raa001 **EQ_bb**<br>(0.15) [0.18]	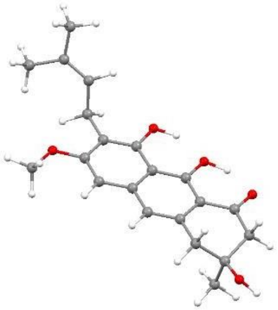<br>Sbb_Raa215 **AX_bb**<br>(0.08) [0.06]	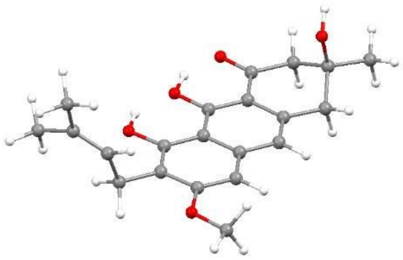<br>**EQ_ba**<br>(0.21) [0.18]	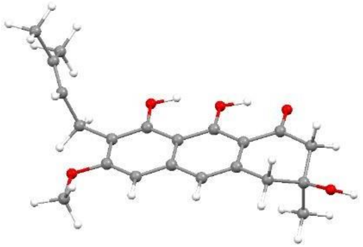<br>**AX_ba**<br>(0.09) [0.07]
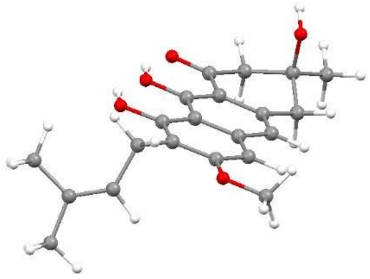<br>**EQ_ab**<br>(0.16) [0.19]	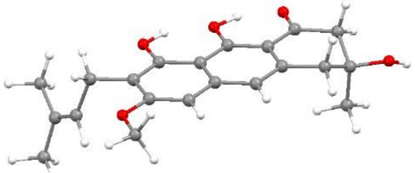<br>**AX_ab**<br>(0.08) [0.07]	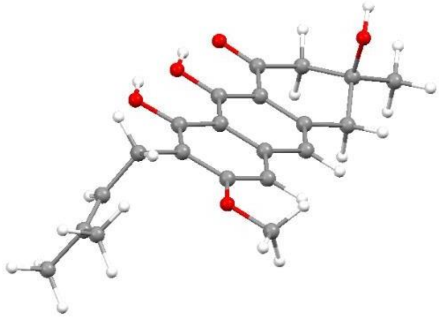<br>**EQ_aa**<br>(0.13) [0.18]	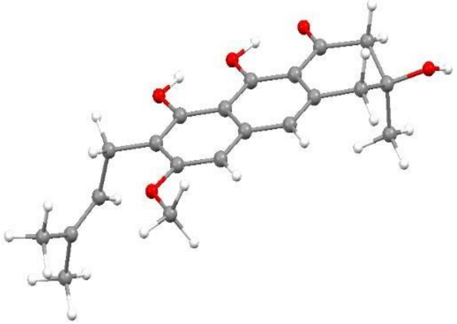<br>**AX_aa**<br>(0.10) [0.07]

The calculated statistical weights of conformations are:

- B3PW91/6-311++G(d)_PCM method = round brackets;
- B3LYP/cc-pvTz_PCM method = square brackets.

According to these data, the total amount of "EQ" conformations is nearly twice as much as is the total amount of "AX" conformations.

Figure S21. ECD spectra for main conformations of 6*S*-1

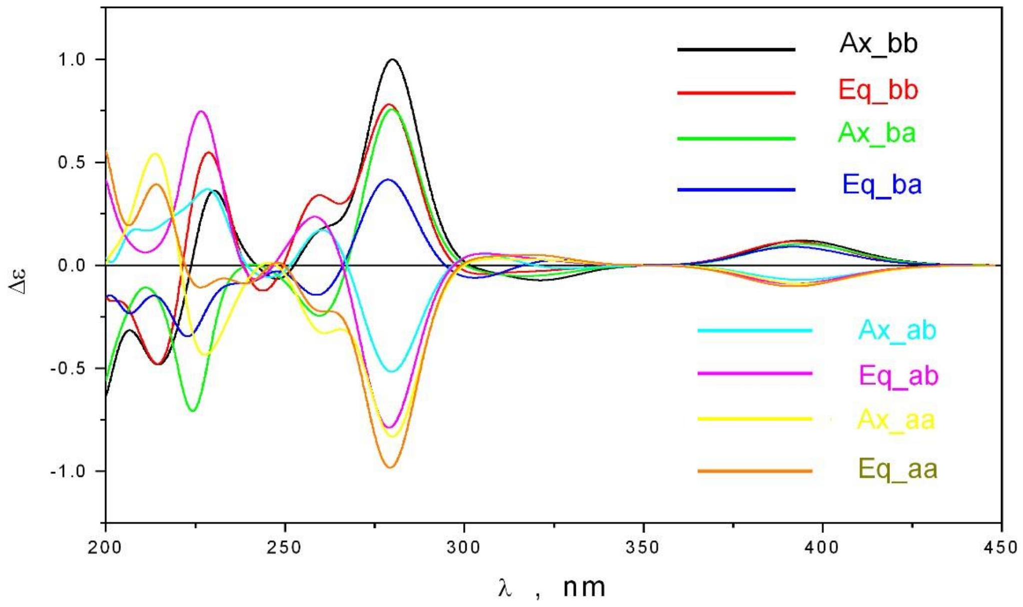

Figure S22. Contribution of main conformations to total ECD spectrum of 6*S*-1

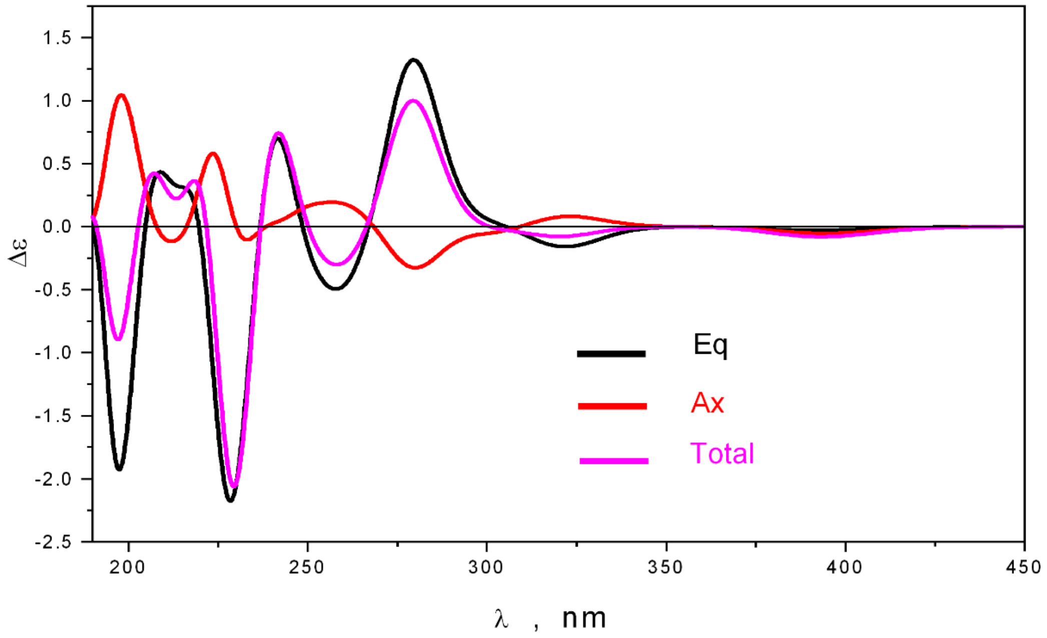

Figure S23. MS/MS spectra of HPLC MS peak #5 (endocrocin)

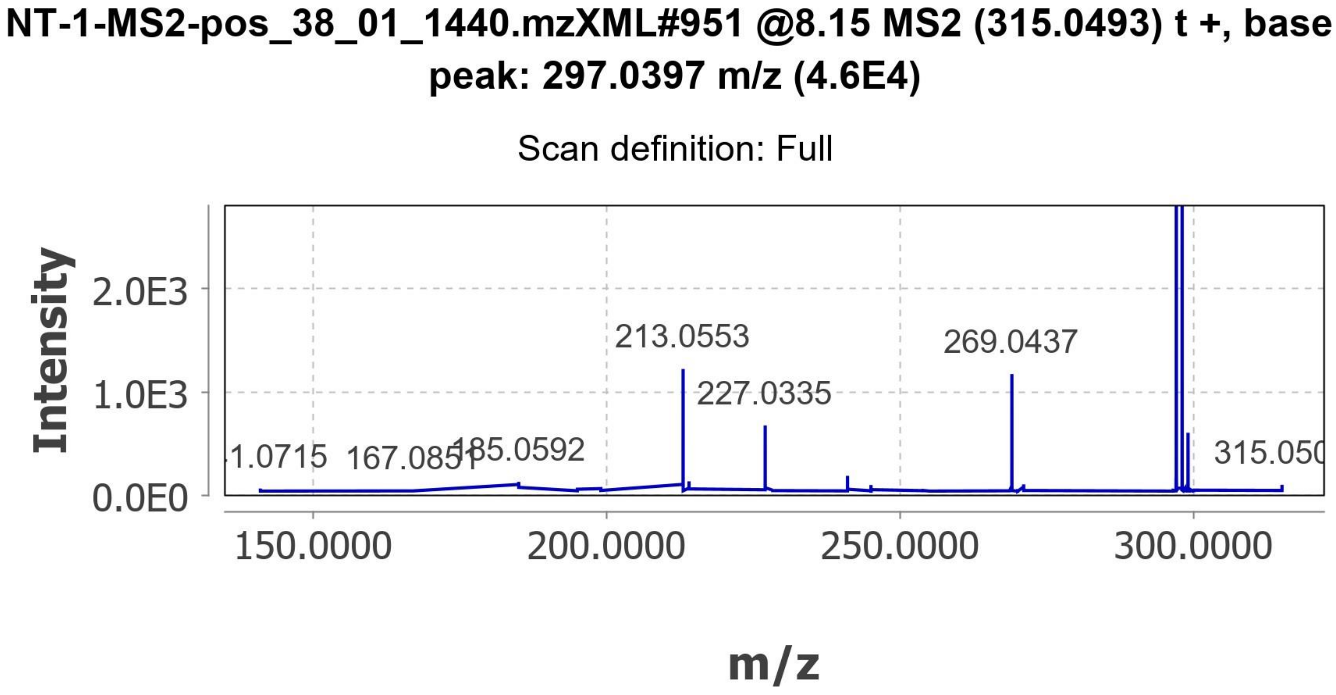

Figure S24. MS/MS spectra of HPLC MS peak #12 (11a-hydroxy-4,4,9-trimethyl-9-vinyl-1,2,3,4,9,10,11,11a-octahydrodibenzo[c,e]oxepine-5,7-dione)

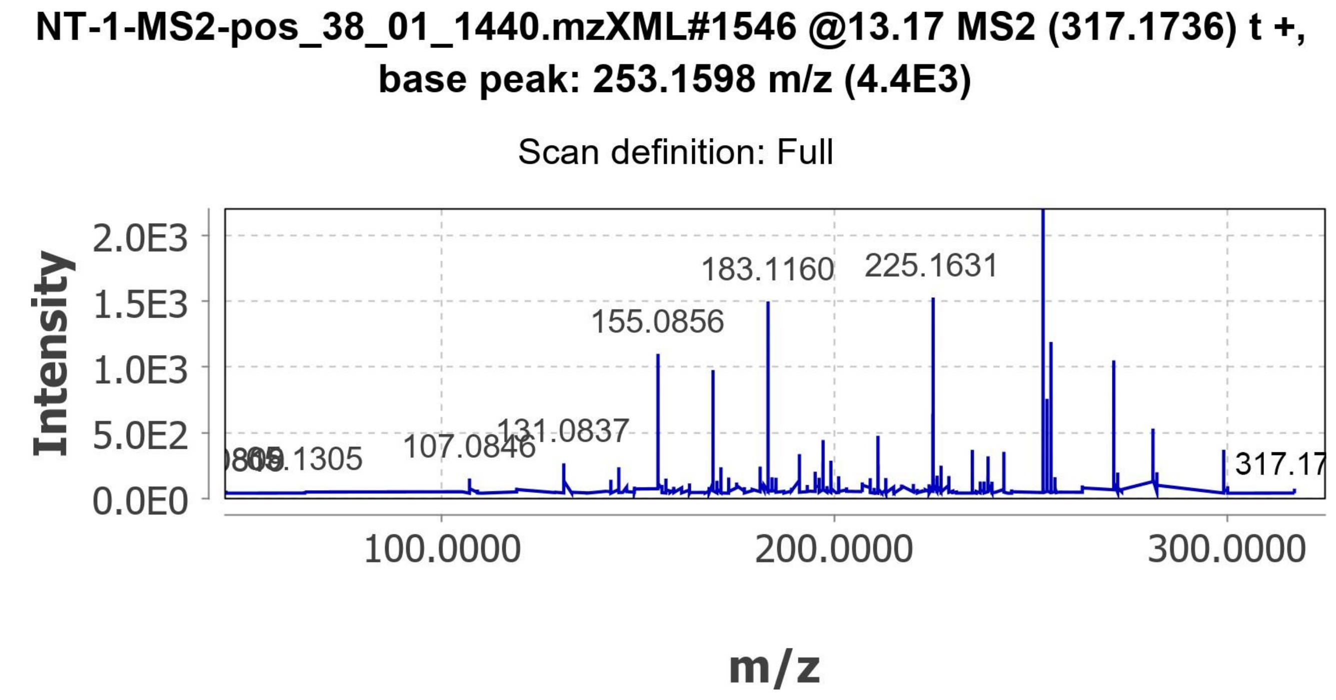

Supplement: Supplementary file 1 [file ijms-24-08150-s001.zip › ijms-2297246-supplementary.pdf]
